# A crucial role for the ubiquitously expressed transcription factor Sp1 at early stages of hematopoietic specification

**DOI:** 10.1242/dev.106054

**Published:** 2014-06

**Authors:** Jane Gilmour, Salam A. Assi, Ulrike Jaegle, Divine Kulu, Harmen van de Werken, Deborah Clarke, David R. Westhead, Sjaak Philipsen, Constanze Bonifer

**Affiliations:** 1 School of Cancer Sciences, Institute of Biomedical Research, College of Medical and Dental Sciences, University of Birmingham, Birmingham B15 2TT, UK; 2 Faculty of Biological Sciences, University of Leeds, Leeds LS2 9JT, UK; 3 Department of Cell Biology, Erasmus MC, Rotterdam 3015 CN, The Netherlands; 4 Section of Experimental Haematology, Leeds Institute of Molecular Medicine, University of Leeds, Leeds LS9 7TS, UK

**Keywords:** Sp1 transcription factor, Hematopoiesis, Transcriptional network, Mouse

## Abstract

Mammalian development is regulated by the interplay of tissue-specific and ubiquitously expressed transcription factors, such as Sp1. *Sp1* knockout mice die *in utero* with multiple phenotypic aberrations, but the underlying molecular mechanism of this differentiation failure has been elusive. Here, we have used conditional knockout mice as well as the differentiation of mouse ES cells as a model with which to address this issue. To this end, we examined differentiation potential, global gene expression patterns and Sp1 target regions in Sp1 wild-type and Sp1-deficient cells representing different stages of hematopoiesis. *Sp1^−/−^* cells progress through most embryonic stages of blood cell development but cannot complete terminal differentiation. This failure to fully differentiate is not seen when Sp1 is knocked out at later developmental stages. For most Sp1 target and non-target genes, gene expression is unaffected by Sp1 inactivation. However, Cdx genes and multiple Hox genes are stage-specific targets of Sp1 and are downregulated at an early stage. As a consequence, expression of genes involved in hematopoietic specification is progressively deregulated. Our work demonstrates that the early absence of active Sp1 sets a cascade in motion that culminates in a failure of terminal hematopoietic differentiation and emphasizes the role of ubiquitously expressed transcription factors for tissue-specific gene regulation. In addition, our global side-by-side analysis of the response of the transcriptional network to perturbation sheds a new light on the regulatory hierarchy of hematopoietic specification.

## INTRODUCTION

Changes in gene expression programs during cell differentiation are regulated by the interplay of ubiquitous and tissue-specific transcription factors, and the epigenetic regulatory machinery. A large number of tissue-specific transcription factors have been described in whose absence a specific cell lineage is not, or is less efficiently, formed. In the hematopoietic system, this includes factors such as GATA1 and PU.1, which are crucial regulators of erythropoiesis or myelopoiesis, respectively (Orkin and Zon, 2008). The effect of removing tissue-specific regulators for a specific pathway leads to dramatic alterations in gene expression and often a block in differentiation at the point where this factor is crucially needed. The reason for this could be that such factors nucleate shifts in transcription factor assemblies to alter cell fates (Heinz et al., 2010; Lichtinger et al., 2012). However, many widely expressed genes are regulated by factors that are also more or less ubiquitously expressed and that cooperate with tissue-specific factors to activate specific gene expression programs. An interesting observation is that knockout of such genes can have surprisingly tissue-specific effects. This is true, for example, for the individual knockouts of NF1 family members (Gronostajski, 2000). A reason for this phenomenon could be compensation by other family members. However, the knockout of factors such as Sp1 or Oct1 arrests development at an early developmental stage (Marin et al., 1997; Sebastiano et al., 2010). We currently know very little of the molecular mechanisms underlying the impact of ubiquitously expressed transcription factors on tissue-specific gene expression. The Sp transcription factor family is a branch of the Krüppel family of zinc-finger proteins (Philipsen and Suske, 1999) with Sp1 as the founding family member. Sp1 is ubiquitously expressed and binds to a GC-rich consensus sequence that is found at most housekeeping genes regulated by CpG island promoters, but also in many tissue-specific genes (Wierstra, 2008). The effect of rendering Sp1 inactive in mice is dramatic. Sp1-deficient embryos die very early in development with variable phenotypes that range from developmental arrest as an amorphous cell mass to retarded embryos with a number of recognizable structures (Marin et al., 1997). Such a pleiotropic phenotype that affects multiple cell types is seen in a number of knockout mice carrying mutations in global regulators, but to actually investigate the molecular mechanisms that underlie how such factors are involved in the regulation of multiple genes in specific tissues is an extremely difficult task. The binding of ubiquitously expressed factors may be to a large extent invariant and the effect of crippling their activity on development may be dictated by tissue-specific factors or by changes in their binding during development. Moreover, the impact of a lack of factor activity on gene expression control during development may be progressive and may only accumulate over many different developmental stages, making it difficult to pinpoint the actual cause of the developmental defect. To date, neither of these scenarios has been investigated.

Here, we address these issues by differentiating mouse embryonic stem cells that carry a DNA binding-deficient Sp1 variant into hematopoietic cells. We purified differentiating cells at successive stages of hematopoietic development and measured global gene expression profiles, as well as differentiation capacity. We also determined the direct target genes of Sp1 using ChIP sequencing. Our data show that *Sp1^−/−^* cells are capable of progressing through all early embryonic stages of blood cell development up to the progenitor stage, but are then unable to progress further. This failure of terminal differentiation is not seen when Sp1 is knocked out at later developmental stages. We demonstrate that the underlying mechanism of this inability to complete differentiation is a progressive deregulation of gene expression over multiple cell generations, with multiple developmental pathways involved in hematopoietic stem cell specification and myeloid differentiation being affected. All four Hox gene clusters, as well as their upstream regulators, the Cdx genes, are targets of Sp1 at an early, but not at a later, differentiation stage and the regulation of a subset of these genes is affected by Sp1 inactivation, providing a molecular explanation for the multiple developmental defects in Sp1-deficient mice.

## RESULTS

### The absence of Sp1 DNA binding activity affects multiple hematopoietic lineages

**Fig. 1.**
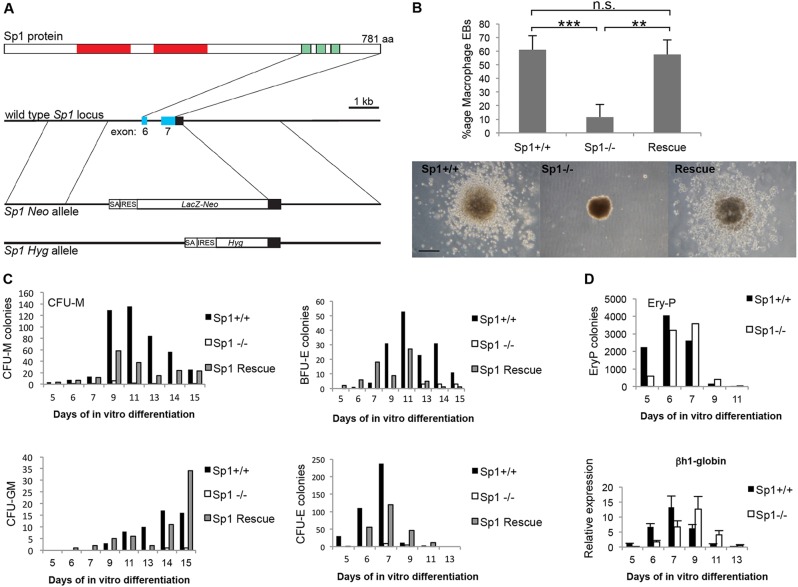
**Absence of Sp1 binding affects the developmental potential of multiple hematopoietic lineages.** (A) The Sp1 deletion mutant. (B) Macrophage release assay. Embryoid bodies were allowed to form in methylcellulose under macrophage-promoting conditions. *Sp1*^−/−^ cells show a reduced capacity to form macrophage-releasing EBs, whereas this capacity is restored in Sp1-rescue cells. *n*=3, error bars represent s.d., **P<0.01, ***P<0.005. (C) Colony assays demonstrating that the re-expression of Sp1 rescues hematopoietic development. Embryoid bodies in methylcellulose were dispersed at different time-points and re-plated in methylcellulose under hematopoietic colony-forming conditions. *Sp1^−/−^* cells show reduced colony forming capacity in all lineages but especially to CFU-M and CFU-GM. A representative graph out of three independent experiments is shown for each colony type. (D) Top: Ery-P colony assay. Embryoid bodies in methylcellulose were dispersed at different time-points and re-plated in methylcellulose supplemented with erythropoietin. Graph shows combined data from two independent experiments performed in duplicate. Bottom: gene expression analysis showing expression of embryonic βh1-globin during EB differentiation, error bars indicate s.e.m. (*n*=3).

In the past decade, a number of attempts have been made to dissect the molecular mechanism of the developmental arrest caused by lack of Sp1 DNA-binding activity, using conditional knockout mice and CRE-recombinase enzyme expressed from different types of tissue-specific promoters. Although such experiments confirmed the severe defects in mice where Sp1 activity was removed in all tissues, other phenotypes were surprisingly mild, if at all visible (D. I. Kulu, PhD Thesis, Erasmus University, Rotterdam, The Netherlands, 2013). This indicates that the timing of the knockout is of essence and that cells have to undergo a number of differentiation stages for it to be visible. Remarkably, ES cells carrying two copies of the mutant Sp1 allele expressing a truncated protein lacking the entire DNA-binding domain (*Sp1*^−/−^; Fig. 1A, supplementary material Fig. S1A) were indistinguishable from wild-type cells in culture, and contributed efficiently to chimeras until E9.5, after which contribution of the mutant cells rapidly declined to undetectable levels (Marin et al., 1997). This opened the possibility of using the differentiation of such cells *in vitro* to obtain molecular insights into the molecular mechanisms of differentiation perturbed by the lack of Sp1 activity. We first tested whether *Sp1*^−/−^ ES cells were capable of differentiating into hematopoietic cells by performing macrophage release assays where ES cells are cultured in the presence of interleukin 3 and colony-stimulating factor 1 (Csf1). This drives the formation of embryoid bodies (EBs) containing macroscopically visible blood islands that release macrophages at later stages of differentiation (Faust et al., 1994; Clarke et al., 2000). *Sp1^−/−^* cells had a greatly reduced ability to form blood islands and macrophages in embryoid bodies compared with wild-type cells (Fig. 1B). Moreover, gene expression analysis with RNA prepared from developing EBs showed reduced levels of mRNA for genes important for myelopoiesis, such as *Spi1* (previously *Sfpi1*, *PU.1*), *Cebpa* and *Csf1r* (supplementary material Fig. S1B). Other hematopoietic lineages, such as erythroid cells, were also affected, as shown by colony assays demonstrating a near complete lack of colony-forming ability (Fig. 1C). This impediment of differentiation was not due to a proliferative defect, as shown by CFSE assays (supplementary material Fig. S1C). We used colony assays to show that mutant phenotypes were a direct result of Sp1 deficiency and not clonal variation of ES cells. Expression of Sp1 cDNA in the same *Sp1^−/−^* clone rescued both macrophage development and colony-forming ability (Fig. 1B,C). However, primitive erythropoiesis producing nucleated erythrocytes occurred at wild-type levels (Fig. 1D and supplementary material Fig. S1D). In addition, embryonic globin was expressed, but was up- and downregulated with delayed kinetics (Fig. 1D and supplementary material Fig. S1D), indicating that this developmental pathway was largely independent of Sp1.

### Hematopoietic development in Sp1-deficient cells is progressively impaired

**Fig. 2.**
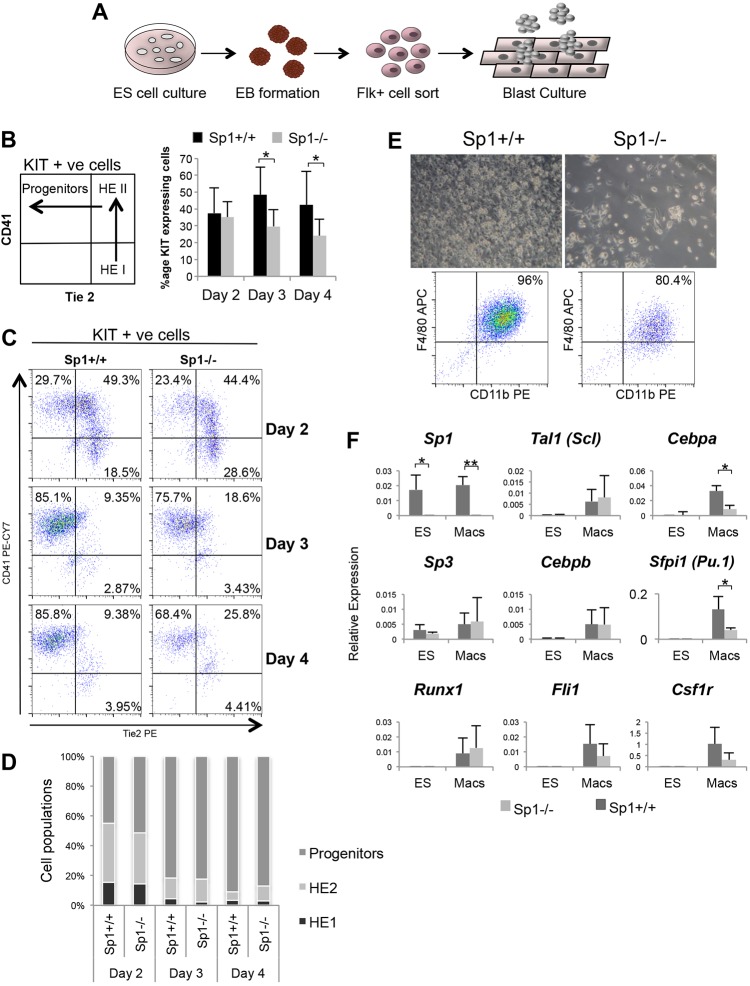
**Abolition of Sp1 DNA binding has a progressively deleterious effect on hematopoietic development.** (A) The hematopoietic differentiation assay used to generate cell populations for microarrays and ChIP-seq. (B) Left: graph depicting FACS analysis of cell populations throughout blast culture differentiation. Cells were stained with antibodies to KIT, CD41 and Tie2. Right: average proportion of KIT-expressing cells as measured by FACS analysis at day 2, 3 and 4 of blast culture (for details, see supplementary material Fig. S2D) (*n*=4, error bars represent s.d., *P<0.05). (C) Representative FACS analysis demonstrating that HE1, HE2 and progenitors are formed in both differentiating wild-type and *Sp1^−/−^* cells, albeit with a slightly reduced frequency. (D) Graphical depiction of C (*n*=4). (E) Differentiation of progenitors to macrophages is dramatically reduced in Sp1 KO cells. Progenitors were seeded under macrophage-promoting conditions. Macrophage markers F4/80 and CD11b were assayed by FACS analysis after 7+ days of differentiation. (F) Gene expression analysis measuring the expression of the indicated hematopoietic regulator genes using RNA prepared from wild-type and *Sp1^−/−^* ES and macrophage cells (*n*=4, error bars represent s.d., *P<0.05, **P<0.01.).

In both ES cells and in the whole organism, hematopoietic cells originate from mesodermal cells that form a precursor with hematopoietic, cardiac and endothelial potential: the hemangioblast (Fehling et al., 2003). This precursor cell type differentiates into specialized adherent endothelial cells forming a hemogenic endothelium (HE), which gives rise to the actual hematopoietic progenitor cells via a two-step process called the endothelial-hematopoietic transition (Chen et al., 2009; Lancrin et al., 2009) (Fig. 2A). Morphological inspection of *Sp1^+/+^* and *Sp1^−/−^* cultures demonstrated they were undistinguishable (supplementary material Fig. S2A) and contained similar numbers of cells (supplementary material Fig. S2B). We also employed a blast colony assay (Kennedy et al., 1997) to show that the development of the earliest stage of hematopoietic development, the hemangioblast, was not disturbed and similar numbers of colonies were formed (supplementary material Fig. S2C). To examine in detail how and at which developmental stage the lack of Sp1 affected hematopoietic development, we measured levels of the stem cell factor receptor KIT, the endothelial marker TIE2 and the integrin CD41 on the cell surface during differentiation by flow cytometry (Fig. 2B-D, supplementary material Fig. S2D). The latter, together with CD45 is a marker of definitive hematopoietic progenitor cells (Mikkola et al., 2003). These experiments show that the proportion of KIT- positive cells and KIT expression levels were progressively reduced during differentiation of *Sp1^−/−^* cells. Interestingly, the proportion of CD45-positive cells was not altered, indicating that HE and progenitor cells were still formed albeit at slightly reduced numbers (supplementary material Fig. S2E). *Sp1^−/−^* progenitors were impaired in colony formation (supplementary material Fig. S2F) and in macrophage development, with only a few adherent cells expressing macrophage surface markers being formed (Fig. 2E). However, the phenotype of these cells was aberrant as the expression of important myeloid regulators in these cells was strongly reduced (Fig. 2F).

We next tested whether we could recapitulate this defect in myeloid maturation by removing Sp1 activity at an early myeloid progenitor stage. To this end, we generated mice carrying two floxed Sp1 alleles on an *Sp3^+/−^* heterozygous background. These mice were crossed with lysozyme-Cre mice, which excise the conditional allele at the common myeloid progenitor stage when lysozyme expression is upregulated (Tagoh et al., 2002). We prepared bone marrow cells from these mice and tested their ability to form colonies and generate macrophages. Supplementary material Fig. S2G shows that, although myeloid colony-forming activity in such progenitors was slightly reduced, they were still capable of efficiently producing macrophages even with the additional reduction of Sp3 levels (supplementary material Fig. S2H) and in spite of high levels of CRE activity (supplementary material Fig. S2I). Taken together, these experiments show: (1) that *Sp1^−/−^* cells are able to progress through multiple differentiation steps but are unable to complete terminal differentiation from myeloid progenitors; and (2) that the effect of Sp1 deficiency is cumulative rather than progenitor type specific.

### Gene expression in differentiating Sp1-deficient cells becomes progressively deregulated

**Fig. 3.**
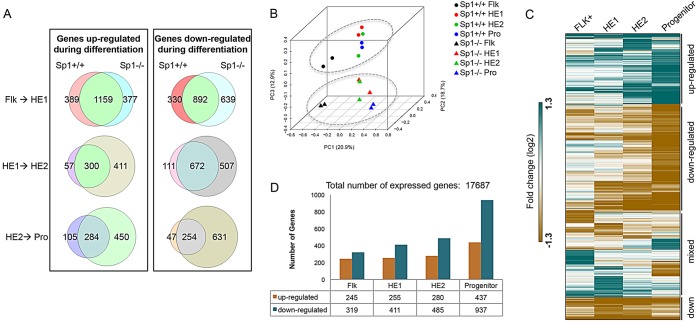
**Gene expression becomes progressively deregulated during hematopoiesis.** (A) Venn diagrams showing the overlap in genes either up- or downregulated during transition from one cell population to the next during differentiation in *Sp1^−/−^* cells relative to wild type. (B) Principal component analysis using the three largest principal components. Although the first two components separate the cell populations, i.e. Flk1^+^, HE1, HE2 and progenitors, the third component separates the wild-type and *Sp1^−/−^* samples, and is indicated by a gray ellipse. (C) Hierarchical clustering of gene expression fold changes for genes that are differentially expressed in *Sp1^−/−^* cells compared with wild type. (D) Graph indicating the numbers of up- and downregulated genes, as defined in C in *Sp1^−/−^* cell populations compared with the same wild-type cell population, indicating that the highest number of deregulated genes is found in the progenitor cell population.

We next examined how Sp1 deficiency deregulated gene expression. To this end, we used the blast culture system to produce pure populations of differentiating cells by cell sorting as previously described (Lancrin et al., 2009; Lichtinger et al., 2012). We isolated Flk1^+^ cells containing hemangioblasts, plated those cells into blast culture and purified two successive stages of hemogenic endothelium development before and after the EHT as well as floating progenitor cells (supplementary material Fig. S3A) using cell sorting as outlined in Fig. 2B. We then prepared RNA from these cells and measured global mRNA expression by microarray analysis (supplementary material Tables S1 and S2). Several hundreds of genes change their expression at each differentiation stage in wild-type cells and this was also true for *Sp1^−/−^* cells (Fig. 3A) whereas the majority of genes in wild-type and *Sp1^−/−^* cells in the different cell populations showed highly similar gene expression profiles (supplementary material Fig. S3B). This was confirmed by Pearson correlation analysis of biological replicates (supplementary material Fig. S3C), indicating a high level of correlation of gene expression profiles between *Sp1^−/−^* and wild-type cells at the start of blast culture in Flk1^+^ cells. However, gene expression patterns progressively deviated during differentiation (compare yellow frames, supplementary material Fig. S3C). This deviation occurred on a background of highly similar gene expression in each cell type, as demonstrated by principal component analysis (Fig. 3B) and analysis of fold-change in gene expression during differentiation (Fig. 3C). Differentiation-directed gene expression changed for most genes as in wild-type cells, indicating little variation between ES cell clones (see also supplementary material Fig. S3C), but a subset of genes was progressively deregulated and wild-type and *Sp1^−/−^* cells could be clearly distinguished. In total, 2220 genes changed expression more than twofold (up or down) as a result of Sp1 deficiency, with 564 deregulated genes in Flk1^+^ cells and 1374 deregulated genes in progenitors (Fig. 3D).

### Sp1 deficiency affects multiple developmental pathways in a cell type-specific fashion

**Fig. 4.**
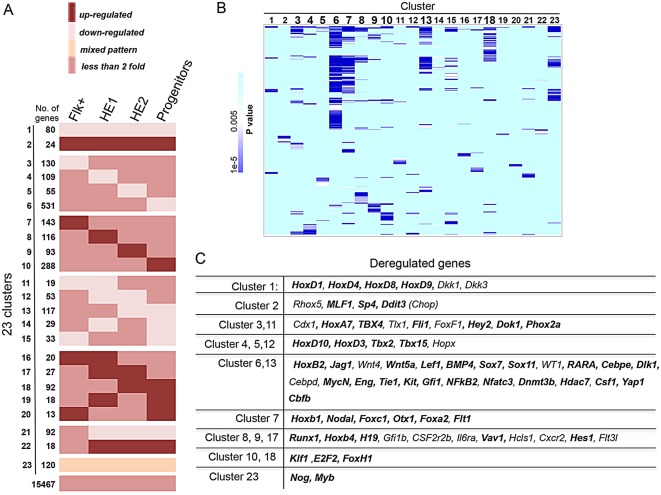
**Analysis of gene expression changes throughout differentiation.** (A) Clustering of genes more than twofold up- or downregulated by Sp1 inactivation during differentiation. Up- and downregulated genes could be separated into 23 clusters according to changes in expression levels during differentiation, as defined in supplementary material Fig. S4A,B. The progenitor population shows the highest number of deregulated genes. (B) Clustering of GO terms after gene ontology analysis of the up- and downregulated genes in *Sp1^−/−^* cells relating to the clustering depicted in A, showing that the different clusters identified are enriched for different patterns of gene ontology terms. (C) List of known developmental regulator genes within the different clusters that are deregulated during the differentiation of *Sp1^−/−^* cells. Direct target genes of Sp1 at any developmental stage are indicated in bold.

In order to identify which developmental pathways were deregulated in *Sp1^−/−^* differentiating cells, we clustered gene expression changes according to patterns of expression throughout differentiation (Fig. 4A, supplementary material Fig. S4A,B and Table S3). This analysis yielded 23 clusters and identified a large number of genes whose expression was unchanged, but also many genes whose expression pattern during differentiation was altered at specific time points of differentiation. Manual validation of expression changes for selected genes by real-time PCR is shown in supplementary material Fig. S4C. In all cell types, most genes were downregulated. Deregulation of gene expression by Sp1 deficiency affected multiple differentiation pathways and gene sets, some of which were common between cell types (Cluster 1, 2), but others were unique for each cell type, as demonstrated by clustering of their GO-terms according to *P* values (Fig. 4B and supplementary material Table S5). Fig. 4C lists selected genes deregulated in *Sp1^−/−^* cells, many of which are known developmental regulators. At early stages of differentiation, expression of genes encoding the caudal related transcription factors *Cdx1* and *Cdx2*, which are known to regulate early hematopoiesis (Wang et al., 2008; Lengerke and Daley, 2012), was reduced. In addition, multiple Hox genes were downregulated. In progenitor cells, Hox gene regulation was also affected but in addition we observed a strong downregulation of multiple genes known to impact on early hematopoietic stem cell specification, including genes in the BMP and Wnt pathways such as *Bmp4*, *Lef1* and *Wnt9a* (Lengerke et al., 2008; Nostro et al., 2008), and also genes that regulate stem cell function (*Myb* and *Fli1*) and myelopoiesis (*Cebpe*) (Verbeek et al., 2001; Mucenski et al., 1991; Spyropoulos et al., 2000). In addition, the expression of *Kit* was downregulated in *Sp1^−/−^* progenitor cells, explaining the reduction of KIT protein on their surface. An interesting finding was that upregulated genes in progenitor cells contained many genes involved in heme biosynthesis and erythroid biology (including *Klf1*, *Gata1* and adult globin genes), in spite of the fact that these cells are unable to form definitive erythroid colonies.

### Sp1 binds to different targets during hematopoietic specification

**Fig. 5.**
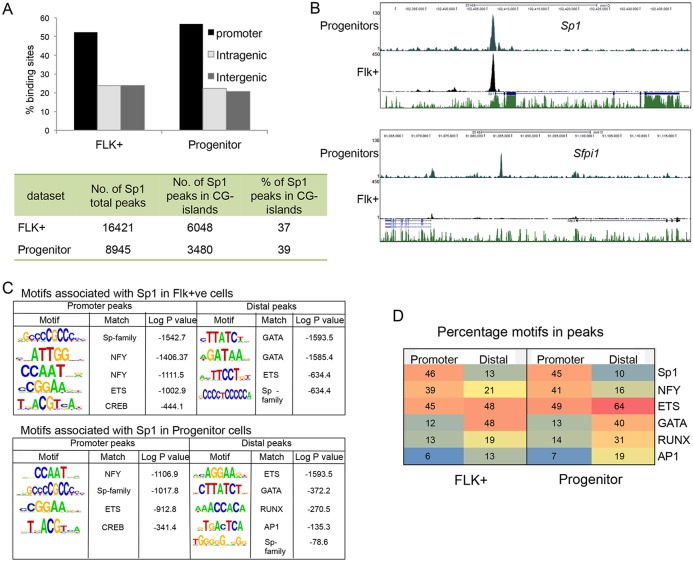
**ChIP-seq analysis of Sp1 binding in wild-type ES, Flk1^+^ and progenitor cells.** (A) Graph showing peak annotations, indicating that the majority of peaks in Flk1^+^ and progenitor cells are in promoter regions. Table shows the percentage of Sp1-binding sites that are within CpG islands for each data set. (B) Screenshots showing examples of Sp1 binding at target loci. Shown are the *Sp1* locus, where Sp1 binds the promoter in both cell populations, and the *Spi1* (*PU.1*) locus where Sp1 binding increases as differentiation progresses. (C) Sp1 colocalizes with motifs for ubiquitously expressed factors at promoters and with motifs for tissue-specific factors at distal elements. Analysis of enriched binding motifs associated with regions of Sp1 binding in each of the cell populations in promoter and distal sites. This shows that motifs associated with the promoter remain relatively consistent, whereas motifs associated with distal binding sites change as differentiation progresses. (D) Table showing percentage of promoter and distal peaks that have the indicated motifs in Flk1^+^ and progenitor populations ±200 bp from peak center.

We next performed ChIP-sequencing in wild-type Flk1^+^ cells and progenitor cells to test which of the deregulated genes were direct targets of Sp1 and to examine whether Sp1 shifted position during differentiation and targeted different genes. Sp1 bound to a large number of sites (supplementary material Fig. S5A), most of which were promoters (Fig. 5A). As predicted from *in vitro* experiments, more than one-third of Sp1-binding sites were located in CG islands (Fig. 5A, bottom panel). The comparison of binding sites between Flk1^+^ cells and progenitors demonstrated that Flk1^+^ cells contained twice as many binding sites as progenitor cells and the expression level of Sp1 in the two cell types differed slightly (supplementary material Fig. S5B). There was a large overlap between binding sites (supplementary material Fig. S5C), and most of these sites (58%) coincided with CG islands, indicating that Sp1 is indeed involved in the regulation of ubiquitously expressed genes. By contrast, the Flk1^+^ and the progenitor-specific peaks contained only 6% and 18% CG islands, respectively. However, there were also many cell type-specific sites, even when only peaks with high tag count were compared (supplementary material Fig. S5D). Such differential binding is exemplified in Fig. 5B, which shows a screenshot of the *Sp1* locus itself that was bound in both Flk1^+^ cells (upper panel) and progenitors compared with the *Spi1* (*PU.1*) locus, which was only bound by Sp1 in progenitors (lower panel). Other examples were the four Hox gene clusters (A-D), which contained a large number of binding sites across each cluster and were specifically bound in Flk1^+^ cells (supplementary material Fig. S5F), indicating that Sp1 cooperates with different factors at tissue-specific genes. This notion was confirmed by analyzing enriched motifs surrounding Sp1 peaks (Fig. 5C, supplementary material Fig. S5E). At promoters, Sp1 colocalized with binding motifs of widely expressed factors such as NFY and CREB in both Flk1^+^ cells and progenitors. However, at distal elements it colocalized with different types of motifs, reflecting the activity of tissue-specific distal elements, with GATA motifs being prominent in Flk1^+^ cells, and highlighting the important role of GATA2 at this stage (Lugus et al., 2007). Sp1 also colocalized with RUNX/ETS/GATA motifs, reflecting the importance of these factors in driving hematopoietic development (Wilson et al., 2010). Approximately half of the promoter sites contained Sp1 consensus motifs, but only a small fraction of the distal sites, indicating the possibility that such peaks originate from the association of Sp1 with upstream factors (Fig. 5D).

**Fig. 6.**
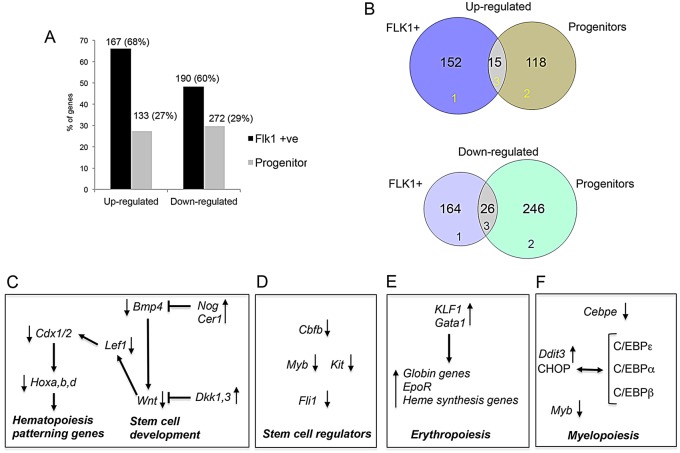
**Integration of ChIP-seq and microarray data.** (A) Graph showing the percentage of genes that are up- or downregulated in *Sp1^−/−^* cells in Flk1^+^ and progenitor cells and that are Sp1 target genes. A hyper-geometric distribution was used to calculate the significance of enrichment of direct Sp1 target genes in Flk1+ and progenitor cells. Enrichment of both up- and downregulated Sp1 target genes was found to be significant, with *P*-values of 4.7e–133 and 3.6e–208 in Flk1^+^ cells and 4.3e–18 and 9.3e–46 in progenitor cells, respectively. (B) Venn diagrams showing the overlap between Sp1 target genes from the ChIP-Seq versus differentially expressed genes in Flk1+ and progenitor cells (upregulated, upper panel; downregulated, lower panel). (C) Schematic outline of the interaction between genes and factors during hematopoietic specification from mesoderm depicting the downregulation of the BMP/Wnt pathway after Sp1 inactivation. (D) Downregulation of stem cell regulators after Sp1 inactivation. (E) Upregulation of regulators of erythropoiesis and globin expression and heme biosynthesis after Sp1 inactivation. (F) Downregulation of myeloid regulators and upregulation of CHOP, which inhibits C/EBP activity after Sp1 inactivation.

The integration of gene expression and ChIP-seq data showed that the majority of Sp1 binding sites were found at genes that were unaffected by the absence of Sp1 activity (supplementary material Fig. S6A). However, in Flk1+ cells more than two-thirds of the deregulated genes were direct Sp1 targets (Fig. 6A, supplementary material Fig. S6A) but this was true for only one-third of the deregulated genes in progenitor cells. There was little overlap between the two gene sets, again showing that Sp1 deficiency affected different gene sets depending on the cellular context (Fig. 6B, see also supplementary material Fig. S6B). A summary of all affected pathways in the different populations is depicted in supplementary material Fig. S7A-E. We found that deregulated Sp1 target genes were involved in multiple pathways, including metabolism, focal adhesion and signaling processes. Furthermore, genes involved in early hematopoietic specification and stem cell development pathways were also affected, and are highlighted in more detail in Fig. 6 and supplementary material Fig. S7.

Our analysis uncovered a clear hierarchy of progressive deregulation events impacting on hematopoiesis. The four Hox clusters A-D and *Cdx1* were major Sp1 targets in Flk1+ cells (supplementary material Table S4), but not in progenitor cells (Fig. 4C, supplementary material Fig. S5F), indicating that Sp1 activity is directly required for correct activity of early regulators of hematopoietic specification at this specific developmental stage (Fig. 6C). However, Sp1 did not only act as an activator, but also as repressor as many targets were upregulated in *Sp1^−/−^* cells (supplementary material Table S4). Two of the most interesting of these genes were *Nog* and *Cer1*, which encode inhibitors of BMP signaling (Smith et al., 1993) with the latter being upregulated 11-fold (supplementary material Table S1), reinforcing the idea that the BMP4/WNT signaling pathway is impaired in the absence of Sp1 DNA-binding activity. This pathway was also affected in progenitor cells (Fig. 4, cluster 6, 13), with a further reduction of *Bmp4*, *Lef1* and *Cdx1/3* expression (supplementary material Table S4).

In progenitors, downregulated Sp1 targets also contained a large number of genes known to be crucial for correct stem cell formation and myeloid differentiation (Fig. 4, supplementary material Table S4). This included transcription factor genes *Myb*, *Fli1*, *Cebpe* and *Cbfb*. *Cbfb* encodes the binding partner (CBFβ) for the master regulator of stem cell development RUNX1 (North et al., 1999) (Fig. 6D, supplementary material Table S4). Again, also in this cell type, Sp1 appeared to be able to act as a repressor, as evidenced by the upregulation of a number of genes, including the WNT inhibitors *Dkk1* and *Dkk3*, as well as *Klf1* and *Ddit3* (Fig. 6E,F)*. Klf1*, which encodes the transcription factor KLF1 (EKLF), is an important activator of erythroid genes and controls multiple aspects of terminal erythropoiesis (Tallack et al., 2010; Pilon et al., 2011). In addition to KLF1, another important erythroid regulator, GATA1, was also upregulated in *Sp1^−/−^* progenitors, which together may explain the upregulation of genes involved in heme biosynthesis as well as fetal and adult globin genes. *Ddit3* was upregulated more than fourfold (supplementary material Table S2) and encodes the C/EBP family member CHOP, which has been shown to repress the transcriptional activity of other C/EBP proteins (Ron and Habener, 1992). C/EBP family members, in particular C/EBP α and ε are essential for myelopoiesis beyond the GMP stage (Friedman, 2007), which may provide one explanation why this differentiation pathway is blocked later than the multipotent progenitor stage.

Collectively, we conclude that the initial downregulation of the Sp1 target loci Cdx and Hox is followed by progressively deregulated expression of genes involved in the control of hematopoiesis over multiple differentiation stages. These early deregulation events set a cascade in motion that culminates in a catastrophic failure of terminal differentiation.

## DISCUSSION

In this study, we perturbed hematopoietic development by inactivating a single, ubiquitously expressed transcription factor (Sp1) and analyzed the effects of this perturbation on the dynamics of differential gene expression and cell differentiation. In addition to dissecting a so far unknown role of Sp1 in blood cell development, our study uncovered a number of novel aspects of the hierarchical control of blood cell development and homeostatic mechanisms maintaining cellular function.

### Sp1 binds to developmental regulator genes in a cell-type specific fashion

Fifty percent of Sp1-binding sites reside in promoters, one-third of which are CG islands, many of which are shared between the two cell types analyzed. Sp1 differentially occupied distal cis-regulatory elements in Flk1^+^ cells and progenitors, and colocalized with different sets of motifs for cell-type specific transcription factors. This cell-type specific occupancy profile was particularly apparent in Flk1^+^ cells with the Hox genes being a prominent example. Many such genes are expressed throughout differentiation, suggesting that at the progenitor stage they are regulated by a set of factors that do not depend on Sp1. The difference in the occupancy profile is reflected in the sensitivity of target genes to Sp1 depletion. The vast majority of genes was not influenced by the absence of Sp1 during cell differentiation, indicating that robust mechanisms exist that compensate for the absence of this protein, even in the face of a changing cis-regulatory environment. Compensating factors are likely to be other Sp-family members such as Sp3 as indicated by the finding that mice heterozygous for either *Sp1* or *Sp3* are viable, but compound heterozygous are not (Kruger et al., 2007). However, the absence of Sp1 strongly affected the expression of distinct subsets of cell type-specific genes, demonstrating that, for such genes, Sp1 cooperates with cell type-specific factors and is a crucial component of stable transcription factor complexes regulating such genes.

The deregulation of gene expression patterns during ES cell-derived hematopoietic differentiation starts with a small number of genes in Flk1^+^ cells, most of which are direct Sp1 targets and this number increases as differentiation progresses, although the proportion of direct targets goes down. Remarkably, although many genes are deregulated in differentiating *Sp1^−/−^* cells and their number increases at later stages of differentiation, many others are not and, up to a point, cell identity is preserved as measured by the ability of these cells to give rise to specific progeny and expressing the majority of genes correctly. Albeit with reduced efficiency, Flk1^+^ cells still differentiate into hemogenic endothelium cells, which are able to undergo the endothelial-hematopoietic transition and thereafter express first CD41 and then CD45, indicating that powerful feedback mechanisms must exist that preserve cellular function. So far the nature of these mechanisms is unknown, but they are likely to involve post-transcriptional regulation mechanisms by, for example, miRNAs, which could buffer the effect of shifts in the transcriptional network on the proteome. Our data clearly show that the deregulation of gene expression after Sp1 inactivation is cumulative and progressive, providing a molecular explanation for: (1) the heterogeneity of the Sp1 knockout phenotype as individual animals will differentially accumulate defects, depending on how well their transcriptional network can compensate; and (2) the lack of effect of knocking out Sp1 at later stages of development.

### Multiple pathways driving hematopoietic development are impaired in Sp1-deficient cells

Our perturbation study also provides profound insights into the hierarchical control of multipotent hematopoietic progenitor cell specification and defines several interlinked pathways involved in the control of this process in an unbiased fashion. This discussion will not be able to cover all aspects of deregulation, but will highlight a few important pathways. The first steps towards hematopoiesis involve the interplay of BMP and WNT signaling, and the activation of Cdx and Hox genes in cells of mesodermal origin (Lengerke et al., 2008; Nostro et al., 2008; Lengerke and Daley, 2012). Our data show that Sp1 is crucially involved in activating these pathways after the hemangioblast stage (Fig. 6C), as both *Cdx1* and some of its downstream Hox target genes are downregulated in the absence of Sp1. Interestingly, the BMP and WNT inhibitors Noggin/Cerberus and Dickkopf (*Nog*, *Cer1*, *Dkk1*, *Dkk3*) are upregulated, suggesting that they may be part of a negative-feedback loop within this pathway that enforces hematopoietic specification. Although all Hox gene clusters are Sp1 targets, not all of the individual genes responded equally to Sp1 knockout. Of the genes in the HoxA cluster, only *HoxA7* was downregulated more than twofold in Flk1+ cells, which raises the possibility that at this stage this gene may contribute to the defects in hematopoiesis and also to the hematopoietic defects observed in mice lacking the entire *HoxA* cluster (Di-Poi et al., 2010). Interestingly, the *HoxB* cluster responded differentially, with *HoxB4*-*HoxB8* being downregulated and *HoxB1* being upregulated in Flk1^+^ cells, consistent with the role of these genes in blood cell development (Bijl et al., 2006) and also confirming that HOXB3 is a repressor of *HoxB1* (Wong et al., 2011). The *HoxC* cluster was mostly unaffected by Sp1 knockout. The most dramatic effect of Sp1 inactivation on expression was seen with the *HoxD* cluster, where basically all genes except *HoxD13* were strongly downregulated at all developmental stages. So far, a role of HoxD proteins in hematopoiesis has not been described (Alharbi et al., 2013), although a leukemogenic version of *HoxD13* was discovered (Pineault et al., 2003). However, Abd-related *Hox* proteins are able to form heterodimers with another protein, MEIS1 (Shen et al., 1997), which also plays an important role in hematopoiesis (Hisa et al., 2004) and cooperates with Hox genes in leukemogenesis (Argiropoulos et al., 2007). *Meis1* was 1.8-fold downregulated after Sp1 knockdown in Flk1^+^ and progenitor cells (supplementary material Table S1), raising the possibility of a combined negative effect on hematopoietic development.

The downregulation of genes involved in hematopoietic specification has a significant knock-on effect on gene expression at the following differentiation stages and affects a number of well-known hematopoietic regulators. Levels of *Myb*, a gene that is absolutely essential for definitive hematopoiesis (Mucenski et al., 1991) and whose expression levels have to be strictly controlled (Taketani et al., 2002), are reduced throughout differentiation. This is also true for levels of *Fli1*, which is essential for fetal liver hematopoiesis (Spyropoulos et al., 2000) and is required for early priming events in blood cell development (Lichtinger et al., 2012). *Runx1* is prematurely upregulated at the first stage of the hemogenic endothelium (CD41^−^ cells), together with RUNX1-responsive targets *Gfi1* and *Spi1* (supplementary material Table S1) (Lancrin et al., 2012; Lichtinger et al., 2012) which is one of the first signs that hematopoietic differentiation is going astray. A reason for this could be the fact that *HoxA3*, which is specifically expressed at this stage and has been shown to be required for the repression of *Runx1* in the hemogenic endothelium (Iacovino et al., 2011) is 1.6-fold downregulated (supplementary material Table S1). At the next stage, after the endothelial-hematopoietic transition (CD41+ cells), the expression of the erythroid regulators *Klf1* and *Gata1*, as well as expression of embryonic globin genes, increases almost fourfold, whereas expression of genes specifying the myeloid lineage, such as *Spi1* and *Cebpe*, is reduced (although normally expression would increase) (Lichtinger et al., 2012) (supplementary material Table S1). This indicates that developing cells pass through the endothelial-hematopoietic transition with an inherent bias for erythropoiesis and that the timing of RUNX1 expression is of the essence for correct myelopoiesis. This bias is further reinforced by the upregulation of *Ddit3* and is even more pronounced at the progenitor stage, explaining the inability of progenitors to form macrophages.

Our data demonstrate that primitive erythropoiesis is not affected by the lack of Sp1, but is delayed. In addition, the kinetics of globin regulation is altered as embryonic and adult globin genes and the erythropoietin receptor are upregulated at the same time, together with genes coding for multiple components of the heme biosynthetic pathway. However, definitive cells expressing adult globin genes are not functional erythroblasts, as they are unable to form erythroid colonies. This is consistent with the notion that, together with Sp3, Sp1 is required for the regulation of fetal liver erythropoiesis (Kruger et al., 2007).

In summary, our approach of following hematopoietic differentiation of *Sp1^−/−^* ES cells combined with the integration of global RNA expression and ChIP data turned out to be highly useful for dissecting a so far unexplained knockout phenotype. Our strategy may serve as an example on how to delineate molecular explanations for knockout phenotypes of other global regulators that also may be the result of cumulative deregulation events. So far we have focused on pathways known to be involved in hematopoietic specification to build the hierarchies outlined in the discussion and to validate our approach, thereby also discovering novel regulatory interactions. However, it should be noted that additional pathways are affected by Sp1 deficiency (supplementary material Fig. S7). This suggests that our approach could also be used to predict novel regulators of other developmental pathways whose individual role can then be tested experimentally.

## MATERIALS AND METHODS

### ES cell maintenance

*Sp1^+/+^*, *Sp1^−/−^* and Sp1-rescue ES cells (Marin et al., 1997) were maintained on primary mouse embryonic fibroblasts in ES maintenance media: DMEM [high glucose from powder (Sigma D5648)], supplemented with 15% FCS, 1 mM sodium pyruvate, 100 units/ml penicillin and 100 µg/ml streptomycin, 1 mM glutamine, 0.15 mM MTG, 25 mM HEPES buffer, 10^3^ U/ml ESGRO (Millipore) and 1× non-essential amino acids (Sigma). Prior to differentiation, the ES cells were grown without feeder cells for two passages on gelatinized tissue culture-treated plates.

### ES cell differentiation in the blast culture system

*In vitro* differentiation of ES cells was performed essentially as described previously (Lichtinger et al., 2012). A more-detailed description can be found in supplementary Materials and methods.

### Macrophage differentiation and colony assays

#### Macrophage release assays

ES cells were trypsinized and allowed to form embryoid bodies by plating in base methylcellulose (Stem Cell Technologies M3134) supplemented with 10% FCS, 100 units/ml Penicillin and 100 µg/ml Streptomycin, 1 mM glutamine, 0.15 mM MTG, 10 µg/ml insulin (Sigma), 10% M-CSF conditioned media, 5% IL-3 conditioned media, 10 ng/ml recombinant mouse M-CSF (R&D Systems), 100 units/ml IL-1 (Peprotech) at a cell concentration previously determined to give similar numbers of EB. After at least 14 days EB were counted and assessed for the number of EB which were surrounded by a halo of macrophages.

#### Macrophage differentiation from progenitors

Macrophages were generated by plating floating progenitors onto low adherence plates at 0.5-1×10^6^ cells per 3 cm dish in IMDM supplemented with 10% FCS, 100 units/ml Penicillin and 100 µg/ml Streptomycin, 1 mM glutamine, 0.15 mM MTG, 10% M-CSF conditioned media, 5% IL3 conditioned media and 10 ng/ml murine M-CSF (R&D Systems). Macrophages were usually harvested after 7 days by first gently washing the cells with PBS to remove any non-adherent cells and then trypsinizing the adherent layer. Cell purity was checked by FACS for expression of F4/80 (eBioscience 17-4801) and CD11b (eBioscience 12-0112). RNA was prepared by lysing the macrophages in TRIzol.

#### Methylcellulose assays

Methylcellulose assays and RNA preparation from embryoid bodies were performed essentially as described previously (Clarke et al., 2000). For a complete description see supplementary Materials and methods.

### Chromatin immunoprecipitation

Chromatin immunoprecipitation and library preparation were performed essentially as described previously (Lichtinger et al., 2012). For a complete description, see supplementary Materials and methods.

### mRNA expression analysis

RNA was extracted from cells using TRIzol (Invitrogen) according to manufacturer's instruction. First-strand cDNA synthesis was carried out using Superscript II (Invitrogen) according to manufacturer's instructions using 250-500 ng of RNA. Real-time PCR was carried out using ABI SYBR green master mix with 5 µl of diluted cDNA and 0.25 µM primer per 15 µl reaction on an ABI 7900HT machine. Analysis was carried out on samples measured in duplicate. See supplementary material Table S6 for primer sequences. For microarray analysis of sorted populations, a further clean up step was performed using RNeasy Minelute columns (Qiagen).

### Microarrays

Each source sample RNA (25 ng) was labeled with Cy3 dye as per the protocol detailed in the Low Input Quick Amp Labeling Kit (Agilent Technologies – 5190-2305). A specific activity of greater than 6.0 was confirmed by measurement of 260 nm and 550 nm wavelengths with a NanoDrop ND-1000 Spectrophotometer. Labeled RNA (600 ng) was hybridized for 16 h to Agilent SurePrint G3 Mouse 8×60 K microarray slides. After hybridization, slides were washed as per protocol and scanned with a High Resolution C Scanner (Agilent Technologies), using a scan resolution of 3 μm. Feature extraction was performed using Agilent Feature Extraction Software, with no background subtraction.

### Analysis of mice

Bone marrow samples from wild-type Sp1cko/cko::Sp3cko/wt mice and from LysM-cre::Sp1cko/cko::Sp3cko/wt::YFP/wt mice were subjected to clean up using a MACS dead cell removal kit (Miltenyi Biotec). For CFU-C assays, cells were plated at 2×10^4^ cells/ml in Methocult M3434 in duplicate 3 cm dishes and colonies were scored after 10 days. For CFU-Macrophage (CFU-M) assays, cells were plated at 2×10^4^ cells/ml in M3134 methylcellulose (Stem Cell Technologies), supplemented with 10% FCS, 1% BSA, 100 units/ml penicillin, 100 µg/ml streptomycin, 1 mM glutamine, 0.2 mg/ml human transferrin, 10^−4^ M β2-Me, 10 µg/ml insulin (Sigma), 50 ng/ml M-CSF in duplicate 3 cm dishes and macrophage colonies scored after 8 days. Cells were also cultured for expansion of hematopoietic progenitor cells in liquid culture and subsequent differentiation to macrophages. BM cells were cultured in progenitor expansion media [IMDM supplemented with 10% horse serum (Gibco), 100 units/ml penicillin and 100 µg/ml streptomycin, 1 mM glutamine, 20 ng/ml SCF, 10 ng/ml Flt3 ligand and 25 ng/ml TPO] at 1×10^6^ cells/ml. After 2 weeks in culture, progenitors were seeded in macrophage differentiation media as described above.

### Data analysis

Comparison of *Sp1^+/+^*, *Sp1^−/−^* and Sp1-rescue samples was performed using Student's *t*-test. A detailed description of data analyses and more detailed methods can be found in supplementary materials. Data can be accessed at Gene Expression Omnibus (GEO) under the accession number GSE52499.

## Supplementary Material

Supplementary Material
