# Supplementary material for: A crucial role for the ubiquitously expressed transcription factor Sp1 at early stages of hematopoietic specification
Source: Development. 2014 Jun;141(12):2391–401. doi: 10.1242/dev.106054 (PMC4050696; doi:10.1242/dev.106054)
Supplement: Supplementary Material [file supp_141_12_2391__index.html]

A crucial role for the ubiquitously expressed transcription factor Sp1 at early stages of hematopoietic specification — Supplementary Material 

# A crucial role for the ubiquitously expressed transcription factor Sp1 at early stages of hematopoietic specification

## DEV106054 Supplementary Material

**Files in this Data Supplement:**

- **Supplementary Material**
