# Supplementary material for: A crucial role for the ubiquitously expressed transcription factor Sp1 at early stages of hematopoietic specification
Source: Development. 2014 Jun;141(12):2391–401. doi: 10.1242/dev.106054 (PMC4050696; doi:10.1242/dev.106054)
Supplement: Supplementary Material [file supp_141.12.2391_DEV106054.pdf]

## SUPPLEMENTARY MATERIAL

### Supplementary Methods

### ES cell differentiation

ES cells were trypsinised and transferred as a single cell suspension to 15 cm low adherence bacteriological plates (Sterilin) at a concentration of 2.5 X $10^4$ cells/ml in IVD media. IVD media - IMDM supplemented with 15% FCS, 100 units/ml Penicillin and 100 µg/ml Streptomycin, 1 mM glutamine, 0.15 mM MTG, 0.18 mg/ml Human transferrin (Roche 652202) and 50 µg/ml Ascorbic acid. After 3.25-3.75 days embryoid bodies were collected, briefly digested in 1x trypsin/EDTA and gently dissociated. To obtain a single cell suspension cells were passed through a cell strainer and resuspended in IMDM + 20% FCS. Flk1+ve (CD309) cells were isolated using a biotinylated Flk1 antibody (eBioscience 13-5821) used at 5 µl per $10^7$ cells for 15 minutes on ice, followed by 2x wash with MACS buffer (PBS + 0.5% BSA and 2 mM EDTA). Cells bound by the antibody were isolated using MACS anti-biotin beads and MACS LS columns (Miltenyi Biotec) according to the manufacturer's instructions. Isolated Flk1+ve cells were plated in blast media at a concentration of 9-11X$10^3$ cells per $cm^2$ on gelatinized tissue culture treated dishes. Blast media – IMDM supplemented with 10% FCS, 100 units/ml Penicillin and 100 µg/ml Streptomycin, 1 mM glutamine, 0.45 mM MTG, 0.18 mg/ml Human transferrin, 25 µg/ml Ascorbic acid, 20% D4T conditioned media, 5 µg/L mVEGF (Peprotech), 10 µg/L mIL-6 (Peprotech). At Day 2, 3 and 4 surface marker expression was checked by FACS analysis using antibodies to KIT (BD Pharmingen 553356), CD41 (e-bioscience 25-0411) and Tie2 (e-bioscience 12-5987). Cells were also stained with KIT (as above or ebioscience 11-1171-85) in combination with CD34 (553733), CD45 (ebioscience 17-0451-82) or Flk1 (ebioscience 12-5821-83). Sorted populations were prepared at Day 2 of blast culture. Floating and adherent cells were harvested and combined before staining with KIT, CD41 and Tie2 antibodies in MACS buffer. After washing, cells were separated on a Moflow cell sorter into HE1 (KIT+ve, CD41-ve, Tie2+ve), HE2 (KIT+ve, CD41+ve, Tie2+ve) and progenitor (KIT+ve, CD41+ve, Tie2-ve) cell populations according to surface marker expression.

### Colony assays:

ES cells were trypsinised and allowed to form embryoid bodies by plating in base

methylcellulose (Stem Cell Technologies M3134) supplemented with 10% FCS, 100 units/ml Penicillin and 100 μg/ml Streptomycin, 1 mM glutamine, 4.5 x $10^{-4}$M mM MTG, 10 μg/ml insulin (Sigma), 5% IL-3 conditioned media, 10% M-CSF conditioned media, 100 units/ml IL-1 (Peprotech) at a cell concentration previously determined to give similar numbers of EB. EB were harvested at a range of time-points for CFU-C hematopoietic colony assays using Methocult® M3434 complete methylcellulose (Stem Cell Technologies) or for RNA isolation using TRIzol®. Assays were set up by washing out the methylcellulose with PBS, dispersing the EB with collagenase and plating cells at $10^5$/ml in M3434 methylcellulose in duplicate 3cm bacteriological grade dishes. Dishes were scored from Day 3 for CFU-E and from Day 8 for BFU-E, CFU-M and CFU-GM.

Ery-P assays were based on those performed by Sturgeon *et al* and Sroczynska *et al* (Sturgeon et al., 2012) (Sroczynska et al., 2009). Assays were set up using EB at a range of time-points during differentiation as described above and dispersed cells were plated into methylcellulose supplemented with 10% Platelet Derived Serum (Antech), 5% PFHM (Invitrogen), 100 units/ml Penicillin and 100 μg/ml Streptomycin, 2 mM glutamine, 0.18 mg/ml Transferrin, 50 μg/ml ascorbic acid, 4.5 x $10^{-4}$M MTG and 2 U/ml Erythropoietin (R&D Systems) at $10^5$ cells/ml in duplicate 3 cm bacteriological grade dishes. Ery-P colonies were counted on Day 5 after plating. Staining of nucleated erythroblasts: Ery-P colonies were washed off the dishes with PBS and dispersed. Single cells were cyto-spun onto glass slides, fixed with methanol and stained with Accustain Wright-Giemsa stain (Sigma).

Blast colony assays were performed by seeding 10,000 Flk1+ve sorted cells in base methylcellulose supplemented with 10% FCS, 100 units/ml Penicillin and 100 μg/ml Streptomycin, 25% D4T conditioned medium, 0.18 mg/ml Transferrin, 25 μg/ml ascorbic acid, 4.5 x $10^{-4}$M MTG, 5ng/ml VEGF and 5ng/ml IL-6 in duplicate 3cm bacteriological grade dishes. Colonies were counted after 8 days in culture (Kennedy et al., 1997).

**Chromatin immunoprecipitation:**

Flk1+ve sorted cells and KIT+ve sorted floating progenitors from Day 3 blast culture were used for ChIP-seq analysis. Cells were crosslinked for 10 min at room temperature with 1% formaldehyde (Thermo Scientific) and quenched with 1/10th volume 2 M glycine. Nuclei were prepared essentially as described in Lefevre *et al* 2003, sonicated using a Bioruptor water bath in immunoprecipitation buffer I (25 mM Tris 1 M pH 8.0, 150 mM NaCl, 2 mM EDTA pH 8.0, 1% TritonX-100 and 0.25 % SDS). After centrifugation the sheared 0.5-2 kb

chromatin fragments (1-2 x $10^6$ cells) were diluted with 2 volumes immunoprecipitation buffer II (25 mM Tris pH 8.0, 150mM NaCl, 2 mM EDTA pH 8.0, 1% TritonX-100, 7.5% glycerol) and precipitation was carried out for 2-3 hours at 4°C using 2 µg anti-Sp1 antibody (Santa Cruz sc-17824X) coupled to 15 µl Protein-G dynabeads (Invitrogen). Beads were washed with low salt buffer (20 mM Tris pH 8.0, 150 mM NaCl, 2 mM EDTA pH 8.0, 1% TritonX-100, 0.1% SDS), high salt buffer (20 mM Tris pH 8.0, 500 mM NaCl, 2 mM EDTA pH 8.0, 1% TritonX-100, 0.1% SDS), LiCl buffer (10 mM Tris pH 8.0, 250 mM lithium chloride, 1 mM EDTA pH 8.0, 0.5% NP40, 0.5% sodium-deoxycholate) and TE pH 8.0 containing 50 mM sodium chloride. The immune complexes were eluted in 100 µl elution buffer (100 mM $NaHCO_3$, 1% SDS) and after adding 4 µl 5M sodium chloride and proteinase K, the crosslinks were reversed at 65°C overnight. DNA was extracted using the Ampure PCR purification kit (Beckman Coulter) according to manufacturer's instructions and analysed by qRT-PCR. Libraries of DNA fragments from chromatin immunoprecipitation were prepared from approximately 10 ng of DNA. Firstly, overhangs were repaired by treatment of sample material with T4 DNA polymerase, T4 PNK and Klenow DNA polymerase (all enzymes obtained from New England Biolabs, UK) in a reaction also containing 50 mM Tris-HCl, 10 mM $MgCl_2$, 10 mM Dithiothreitol, 0.4 mM dNTPs and 1 mM ATP. Samples were purified after each step using Ampure PCR purification kit (Beckman Coulter). 'A' bases were added to 3' ends of fragments using Klenow Fragment (3´- 5´ exo-), allowing for subsequent ligation of adapter oligonucleotides (Illumina part #1000521) using Quick T4 DNA ligase. After a further Ampure clean up to remove excess adaptors, fragments were amplified in a PCR reaction using adapter-specific primers (5'-CAAGCAGAAGACGGCATACGAGCTCTTCCGATC*T and 5'-AATGATACGGCGACCGAGATCTACACTCTTTCCCTACACGACGCTCTTCCGATC*T). The libraries were purified and adapter dimers removed by running the PCR products on 2% agarose gels and excising gel slices corresponding to fragments approximately 200-300 bp in size, which were then extracted using the Qiagen gel extraction kit. Libraries were validated using quantitative PCR for known targets, and quality assessed by running 1 µl of each sample on an Agilent Technologies 2100 Bioanalyser. Once prepared, DNA libraries were subjected to massively parallel DNA sequencing on an Illumina Genome Analyzer.

## SDS-PAGE and Western Blotting

Protein extracts were separated on 10 % SDS-PAGE gels and western blots prepared by wet

transfer onto nitrocellulose membrane. Blots were blocked with 5 % milk powder in 0.05 % TBS-Tween and incubated with anti-Sp1 (Millipore 07-645), anti-Sp3 (Santa Cruz sc-644) and anti-GAPDH (abcam ab-8245) antibodies. Proteins were visualised using Pierce SuperSignal West Pico Chemiluminescent substrate (Thermo Scientific).

**CFSE Assay**

Single cell suspensions of ES cells at a concentration of 2 $x10^6$ cells/ml were labelled with 1μM CFSE in PBS for 10 min at room temperature. The CFSE staining was quenched with media and cells washed thoroughly to remove excess CFSE. Cells were seeded on gelatinised 12 well TC plates at a concentration of 4 $x10^4$ cells per well in ES cell maintenance media. FACS was performed every 12hrs and unstained cells were used as a negative control.

**Data Analysis**

**Analysis of ChIP-sequencing data**

The raw sequence data in fastq format returned by the Illumina Pipeline was aligned to the mm10 mouse genome build using BWA (Li and Durbin, 2010). The reads in the resulting alignment files in sam format were used to generate density maps using bed-tools (Quinlan and Hall, 2010) and data was displayed using the UCSC Genome Browser (Kent et al., 2002).

Regions of enrichment (peaks) of ChIP data were identified using MACS (Zhang et al., 2008) and cisGenome (Ji et al., 2008) software. The resulting peaks common for the two peak calling methods were considered for further analysis. Peak overlaps, gene annotations and CpG island measurements were performed using in-house scripts. Peaks were allocated to genes if located in either their promoters or within the region of 500 bp downstream and 2000 bp upstream of the transcription start sites (TSS), as intragenic if not in the promoter but within the gene body region, or if intergenic, to the nearest gene located within 100 kb. CpG island coordinates were downloaded from the UCSC genome browser and the number of peaks in CpG islands was calculated if the peak summit lies within the CpG island start and end coordinates.

A number of tools are designed for testing for differential binding sites; here we used MAnorm a Bioconductor R package (Shao et al., 2012). We found that the 10577 peaks unique for FLK1+ cells were statistically significant differential binding sites at a cut-off of a p-value of >= 0.5 and that 8136 (77%) unique peaks were statistically significant at a cut-off of a p-value of >= 0.1. We also found that the 3099 peaks unique for progenitor were statistically significant differential binding sites at a cut-off of a p-value of >= 0.5 and that 2368 (76%) progenitor unique peaks are statistically significant at a cut-off of a p-value of >= 0.1. Moreover 87% of the FLK1+ peaks were differential binding sites by comparing the 10577 unique FLK1+ peaks to the FLK1+ total peaks generated by MACS when using progenitors as a control sample. 77% of unique FLK1+ peaks were statistically significantly differential binding sites at a MACS FDR cut-off of 7.

De novo motif analysis was performed on promoters and non-promoter (distal) peaks separately using HOMER (Heinz et al., 2010). Motif lengths of 6, 8, 10, 12 and 14 bp were identified in within ± 100 bp from the peak summit and a random background sequence option was used. The motif matrices generated by HOMER were scanned against JASPAR with the use of STAMP to identify similarity to known transcription factor binding sites (Mahony and Benos, 2007). The top enriched motifs with a significant log p value score were recorded. The annotatePeaks function in HOMER was used to find occurrences of motifs in peaks and distribution of motif density around the peak summit. In this case we used the discovered motif position weight matrices (PWM) with the most significant log p value.

**Analysis of microarray data**

The microarray gene expression scanned images were analysed with Feature Extraction Software 10.7.1.1 (Agilent) (protocol GE1_107_Sep09, Grid: 028005_D_F_20100614 and platform Agilent SurePrint G3 Mouse GE 8x60K). The raw data output by Feature Extraction Software was analysed using the LIMMA R package (Smyth et al., 2005)with quantile normalisation and background subtraction with the normexp method (Ritchie et al., 2007) using an offset value of 16. Contrast matrix and eBays function were used and p value <= 0.01 was applied. Only genes with a minimum log2 intensity value equal to or greater than 6 in at least one array were selected as expressed genes. Genes that changed expression at least two fold up or down with respect to $Sp1^{-/-}$ were considered as differentially expressed.

The Principal Component Analysis (PCA) was carried out on the average value of the probe set intensity within each experiment and was calculated based on a pair-wise Pearson

correlation coefficient matrix using R (R Core Team, 2013).

Clustering of gene expression was carried out on signal intensity for all expressed genes and on fold-changes for genes associated with at least a two-fold change. Hierarchical clustering was used with Euclidean distance and average linkage clustering. Heatmaps were generated using Mev from TM4 microarray software suite (Saeed et al., 2006). We then clustered/grouped gene expression fold changes according to patterns of expression throughout differentiation (Fig. 4A). This analysis yielded 23 clusters and identified a large number of genes whose expression was unchanged. Signal intensity and fold changes of each cluster/group individually were hierarchically clustered and box plotted respectively. (Figs S4A, B)

Gene ontology (GO) analysis was performed using bingo (Maere et al., 2005) and David online tool at david.abcc.ncifcrf.gov (Huang da et al., 2009). Non redundant GO terms were filtered using REVIGO online tools at (http://revigo.irb.hr) with simRel as a similarity measure and a medium allowed similarity. KEGG Pathway network analysis was performed using ClueGO tools (Bindea et al. 2009) with kappa score = 0.3.

**Supplementary tables:**

**Table 1: List of differentially regulated genes (wt versus Sp1-/-)**

**Table 2: List of genes in 23 clusters and their GO terms**

**Table 3: List of Sp1 target genes in Flk1+ cells and progenitors and their GO terms**

**Table 4: List of Sp1 bound genes that change expression and their GO terms.**

**Table 5: Hierarchical ranking of GO terms for the selected clusters according to pValue.**

**Table 6: Primer List for RT-PCR**

| Gene | Forward Primer | Reverse Primer |
|---|---|---|
| Sp1 | TCATATTGTGGGAAGCGCTTT | CAGGGCAGGCAAATTTCTTCT |
| Sp3 | CGACAGTCCTGCAGATATTAGGATC | AGGTCATTGGTGTTCAGTGTAGAGTC |
| Sp1(human) | GCGAGAGGCCATTTATGTGT | GGCCTCCCTTCTTATTCTGG |
| Runx1 | GCAGGCAACGATGAAAACTACTC | CAAACCTGAGGTCGTTGAATCTC |

| | | |
|---|---|---|
| Tal1 (Scl) | CAACAACAACCGGGTGAAGA | ATTCTGCTGCCGCCATCGTT |
| Sfpi1(Pu.1) | CCATAGCGATCACTACTGGGATTT | TGTGAAGTGGTTCTCAGGGAAGT |
| Cebpα | GCAGGAGGAAGATACAGGAAGCT | ACACCTAAGTCCCTCCCCTCTAAA |
| Cebpβ | GTTTCGGGACTTGATGCAATC | CGCAGGAACATCTTTAAGTGAT |
| Fli1 | TCGTGAGGACTGGTCTGTATGG | GCTGTTGTCGCACCTCAGTTAC |
| Csf1R | CTTTGGTCTGGGCAAAGAAGAT | CAGGGCCTCCTTCTCATCAG |
| Cdx1 | CACAGAGCGGCAGGTAAAGA | GGCCAGCATTAGTAGGGCAT |
| Cdx2 | AGGAGGAAAAGTGAGCTGGC | TTGGCTCTGCGGTTCTGAAA |
| BMP4 | GAGCCAACACTGTGAGGAGT | ATACGGTGGAAGCCCTGTTC |
| Wnt4 | GCCATCGAGGAGTGCCAATA | GCCACACCTGCTGAAGAGAT |
| HoxA7 | AGCCAGTTTCCGCATCTACC | CCTTCTCCAGTTCCAGCGTC |
| HoxB4 | ACGGCCTACACTCGCCAG | GCGGTTGTAGTGAAACTCCTTCTC |
| HoxD1 | CCACAGCACTTTCGAGTGGA | TGGTGCTGAAATTTGTGCGG |
| Lef1 | ACGACAAGGCCAGAGAACAC | CATGTACGGGTCGCTGTTCA |
| βh1-globin | GGGTTAAGAACATGGACAACCTC | GGGTGAATTCCTTGGCAAAATGA |
| Hba-X | ATCATCATGTCCATGTGGGAGA | GGAAGTAGGTCTTCGTCTGGG |
| Hbb-Y | TGAACTGCACTGTGACAAGC | TGCCGAAGTGACTAGCCAAA |

## Supplementary References:


**Heinz, S., Benner, C., Spann, N., Bertolino, E., Lin, Y. C., Laslo, P., Cheng, J. X., Murre, C., Singh, H. and Glass, C. K.** (2010). Simple combinations of lineage-determining transcription factors prime cis-regulatory elements required for macrophage and B cell identities. *Molecular cell* **38**, 576-89.

**Huang da, W., Sherman, B. T. and Lempicki, R. A.** (2009). Systematic and integrative analysis of large gene lists using DAVID bioinformatics resources. *Nat Protoc* **4**, 44-57.

**Ji, H., Jiang, H., Ma, W., Johnson, D. S., Myers, R. M. and Wong, W. H.** (2008). An integrated software system for analyzing ChIP-chip and ChIP-seq data. *Nat Biotechnol* **26**, 1293-300.

**Kennedy, M., Firpo, M., Choi, K., Wall, C., Robertson, S., Kabrun, N. and Keller, G.** (1997). A common precursor for primitive erythropoiesis and definitive haematopoiesis. *Nature* **386**, 488-93.

**Kent, W. J., Sugnet, C. W., Furey, T. S., Roskin, K. M., Pringle, T. H., Zahler, A. M. and Haussler, D.** (2002). The human genome browser at UCSC. *Genome research* **12**, 996-1006.

**Li, H. and Durbin, R.** (2010). Fast and accurate long-read alignment with Burrows-Wheeler transform. *Bioinformatics* **26**, 589-95.

**Maere, S., Heymans, K. and Kuiper, M.** (2005). BiNGO: a Cytoscape plugin to assess overrepresentation of gene ontology categories in biological networks. *Bioinformatics* **21**, 3448-9.

**Mahony, S. and Benos, P. V.** (2007). STAMP: a web tool for exploring DNA-binding motif similarities. *Nucleic acids research* **35**, W253-8.

**Quinlan, A. R. and Hall, I. M.** (2010). BEDTools: a flexible suite of utilities for comparing genomic features. *Bioinformatics* **26**, 841-2.

**Ritchie, M. E., Silver, J., Oshlack, A., Holmes, M., Diyagama, D., Holloway, A. and Smyth, G. K.** (2007). A comparison of background correction methods for two-colour microarrays. *Bioinformatics* **23**, 2700-7.

**Saeed, A. I., Bhagabati, N. K., Braisted, J. C., Liang, W., Sharov, V., Howe, E. A., Li, J., Thiagarajan, M., White, J. A. and Quackenbush, J.** (2006). TM4 microarray software suite. *Methods Enzymol* **411**, 134-93.

**Shao, Z., Zhang, Y., Yuan, G. C., Orkin, S. H. and Waxman, D. J.** (2012). MAnorm: a robust model for quantitative comparison of ChIP-Seq data sets. *Genome Biol* **13**, R16.

**Smyth, G. K., Michaud, J. and Scott, H. S.** (2005). Use of within-array replicate spots for assessing differential expression in microarray experiments. *Bioinformatics* **21**, 2067-75.

**Sroczynska, P., Lancrin, C., Pearson, S., Kouskoff, V. and Lacaud, G.** (2009). In vitro differentiation of mouse embryonic stem cells as a model of early hematopoietic development. *Methods Mol Biol* **538**, 317-34.

**Sturgeon, C. M., Chicha, L., Ditadi, A., Zhou, Q., McGrath, K. E., Palis, J., Hammond, S. M., Wang, S., Olson, E. N. and Keller, G.** (2012). Primitive erythropoiesis is regulated by miR-126 via nonhematopoietic Vcam-1+ cells. *Dev Cell* **23**, 45-57.

**Zhang, Y., Liu, T., Meyer, C. A., Eeckhoute, J., Johnson, D. S., Bernstein, B. E., Nusbaum, C., Myers, R. M., Brown, M., Li, W. et al.** (2008). Model-based analysis of ChIP-Seq (MACS). *Genome Biol* **9**, R137.

# Supplementary Figures:

## Supplementary Figure 1.

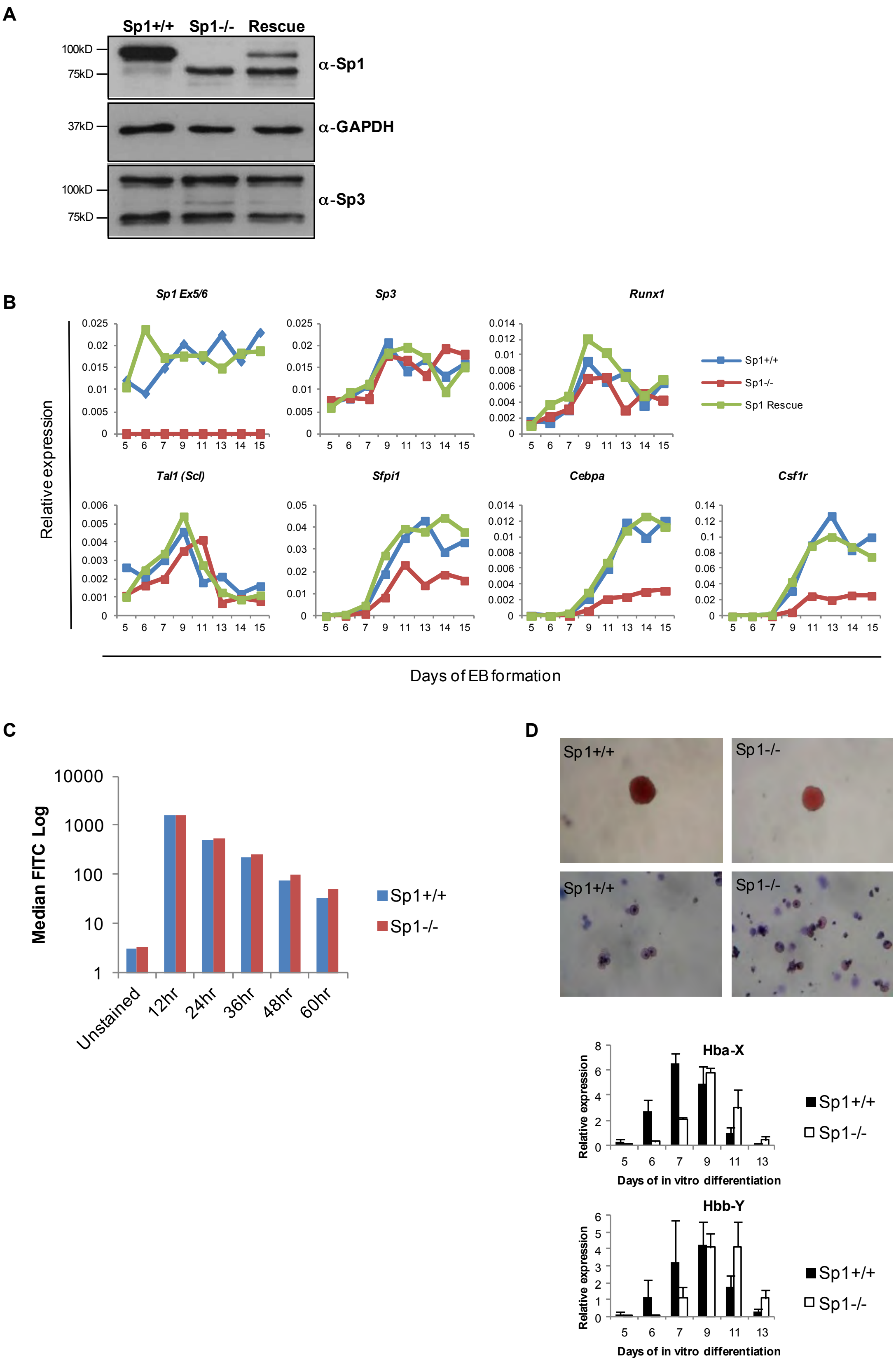

**Supplementary Figure 1**

**A**. Western blot showing the expression levels of WT Sp1 and the deletion mutant in Sp1+/+ wild type, Sp1-/- and Sp1 rescue ES cells. This shows that the Sp1-/- cells do not express the full length Sp1 as they lack the DNA binding domain. Sp1 rescue cells express both the truncated version and the full length Sp1. **B**. Gene expression analysis showing the expression of hematopoietic regulator genes from RNA prepared from embryoid bodies grown in methylcellulose. RNA was taken from EBs used to plate out for the hematopoietic colony assays at various time-points. **C.** CFSE proliferation analysis. Wild-type and Sp1-/- ES cells were labelled with CFSE, plated onto gelatine coated plates and CFSE measured by FACS every 12hrs. **D**. Upper panels: Representative images of individual Ery-P colonies for Sp1+/+ and Sp1-/-, similar morphology was observed. Middle panels: Wright-Giemsa staining of dispersed Ery-P colonies showing nucleated erythroblasts in both Sp1+/+ and Sp1-/-. Lower panels: Gene expression analysis of embryonic globins Hba-X and Hbb-Y. Sp1-/- samples show a delay in expression of embryonic globins but these reach similar levels to Sp1+/+.

**Supplementary Figure 2.**

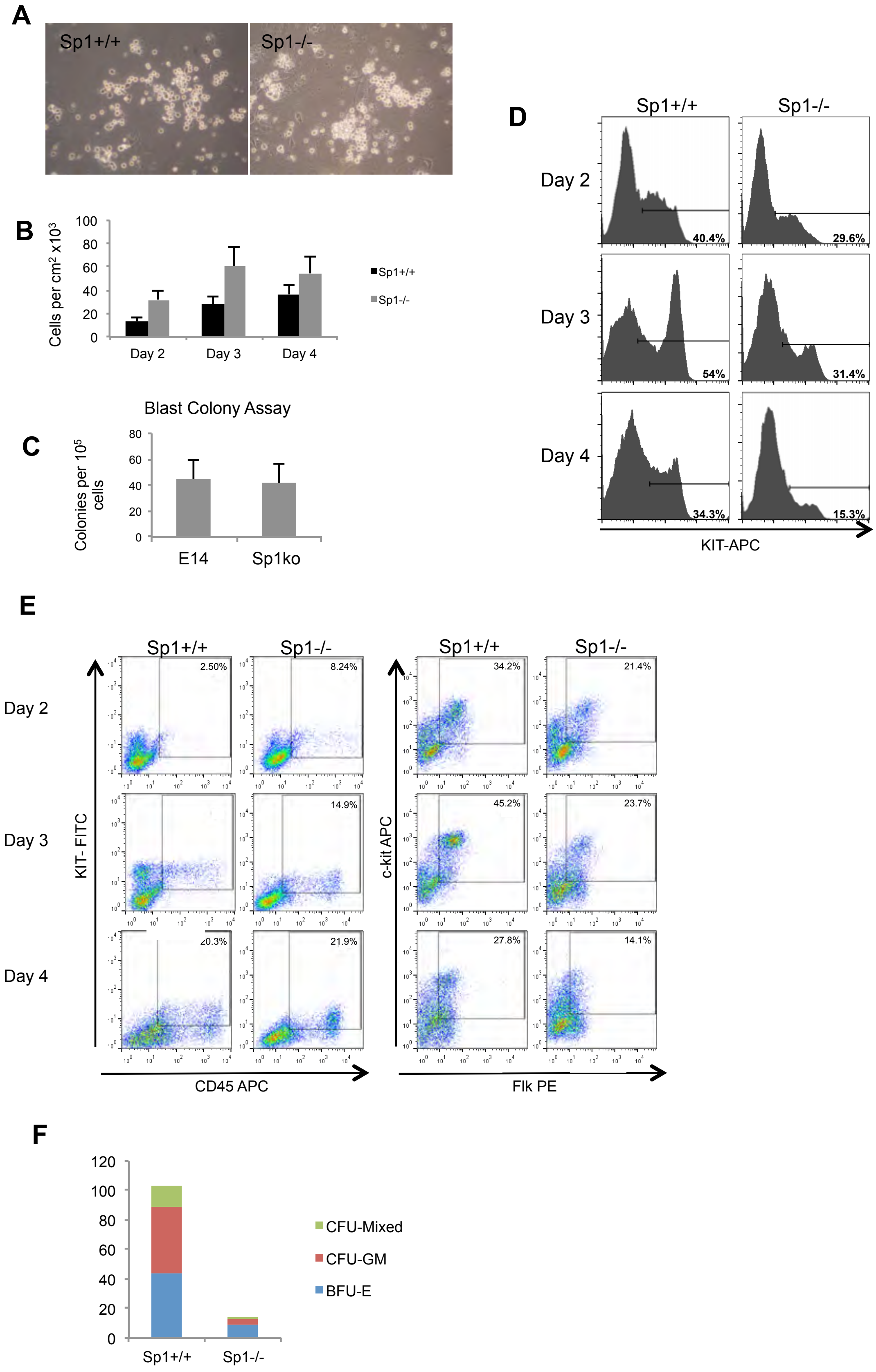

**Supplementary Figure 2. cont'd**

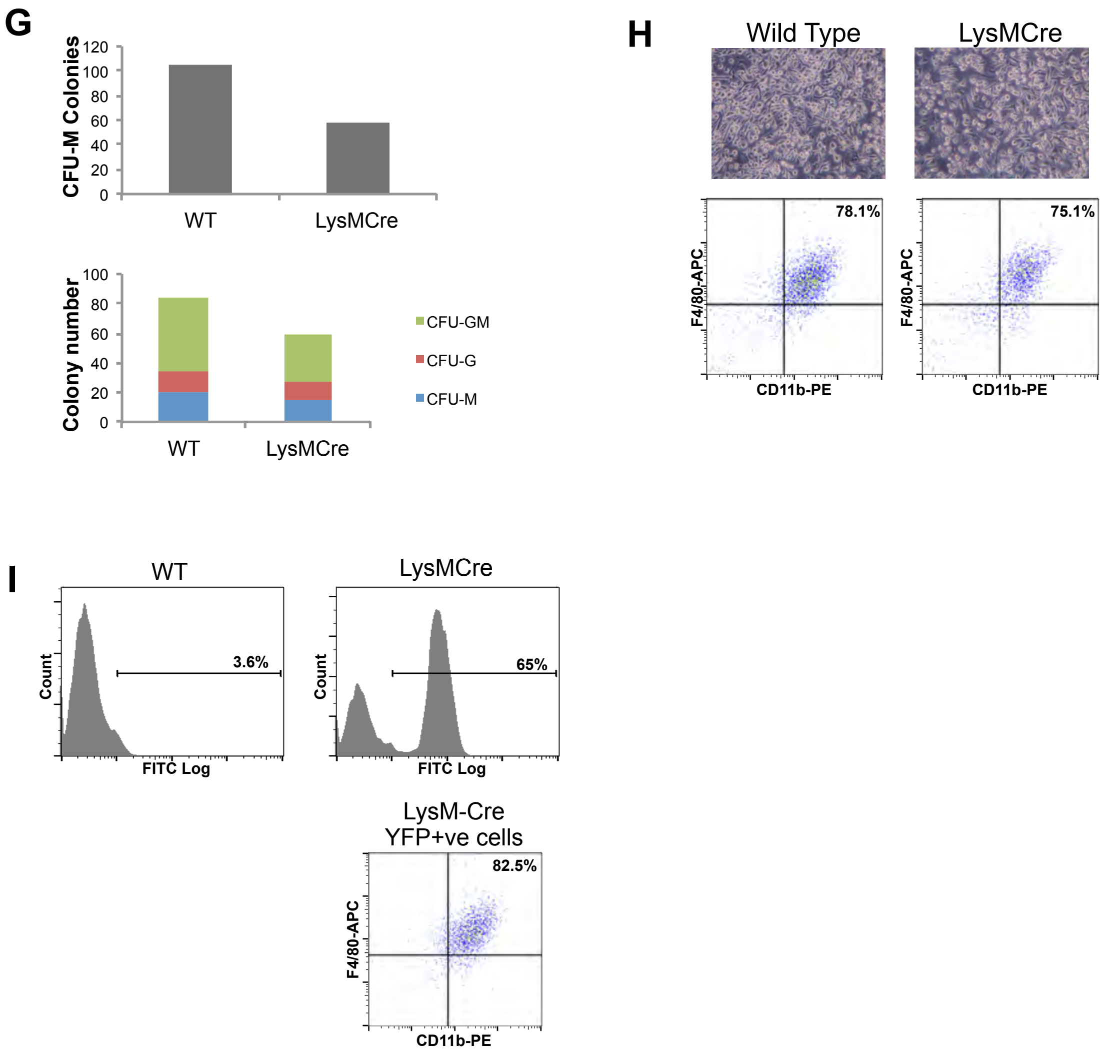


## Supplementary Figure 2.

**A.** Representative images of Day 4 blast cultures of WT and Sp1$^{-/-}$ cells demonstrating that cultures appear morphologically normal. **B.** Cell counts of Sp1+/+ and Sp1-/- cells at days 2, 3 and 4 of blast culture. N=4, no significant difference between Sp1+/+ and Sp1-/- cell counts was observed. **C.** Blast colony assays of Sp1+/+ and Sp1-/- Flk1+ve cells. N=4, no significant difference between Sp1+/+ and Sp1-/- colony formation was observed. **D.** Representative FACS analysis of KIT expression from blast culture differentiation indicates lower levels of KIT expression and a lower proportion of KIT positive cells in Sp1$^{-/-}$ cells (refers to graph in Fig 2B). **E.** Representative FACS analysis of Sp1+/+ and Sp1-/- blast culture populations demonstrating staining of KIT

in combination with CD45 (left) and Flk1 (right) at days 2, 3 and 4 of differentiation. **F**. Colony assays from Day 3 progenitors. Colonies were scored from Day 8, and numbers of all colonies were shown to be reduced in the Sp1-/- samples. **G.** Sp1 deletion at the progenitor stage only slightly reduces myeloid colony formation. Colony assays from cells from Sp1fl/fl/Sp3+/fl x LysMCre and WT bone marrow samples. Dead cell removal was performed on frozen bone marrow cells from WT and conditional deleted mice. Cells were plated out for CFU-M (top) and CFU-C (bottom) colony assays. **H.** Sp1 deletion at the progenitor stage does not affect macrophage differentiation. Progenitors were expanded from Sp1fl/fl/Sp3+/fl x LysMCre and WT bone marrow samples and then differentiated to macrophages in liquid culture. Photographs and FACS analysis show that macrophages differentiate normally from LysMCre Sp1-/- cells. **I.** Conditional deletion is effective in macrophages. To ensure that LysMCre has been activated in macrophages, cells were gated on the YFP+ve cells in the LysMCre Sp1-/- which account for approximately 65% of the cells. These were also positive for F4/80 and CD11b markers of macrophage differentiation. This suggests that Cre-mediated deletion of Sp1 binding in developing adult macrophages does not have a major impact on macrophage differentiation.

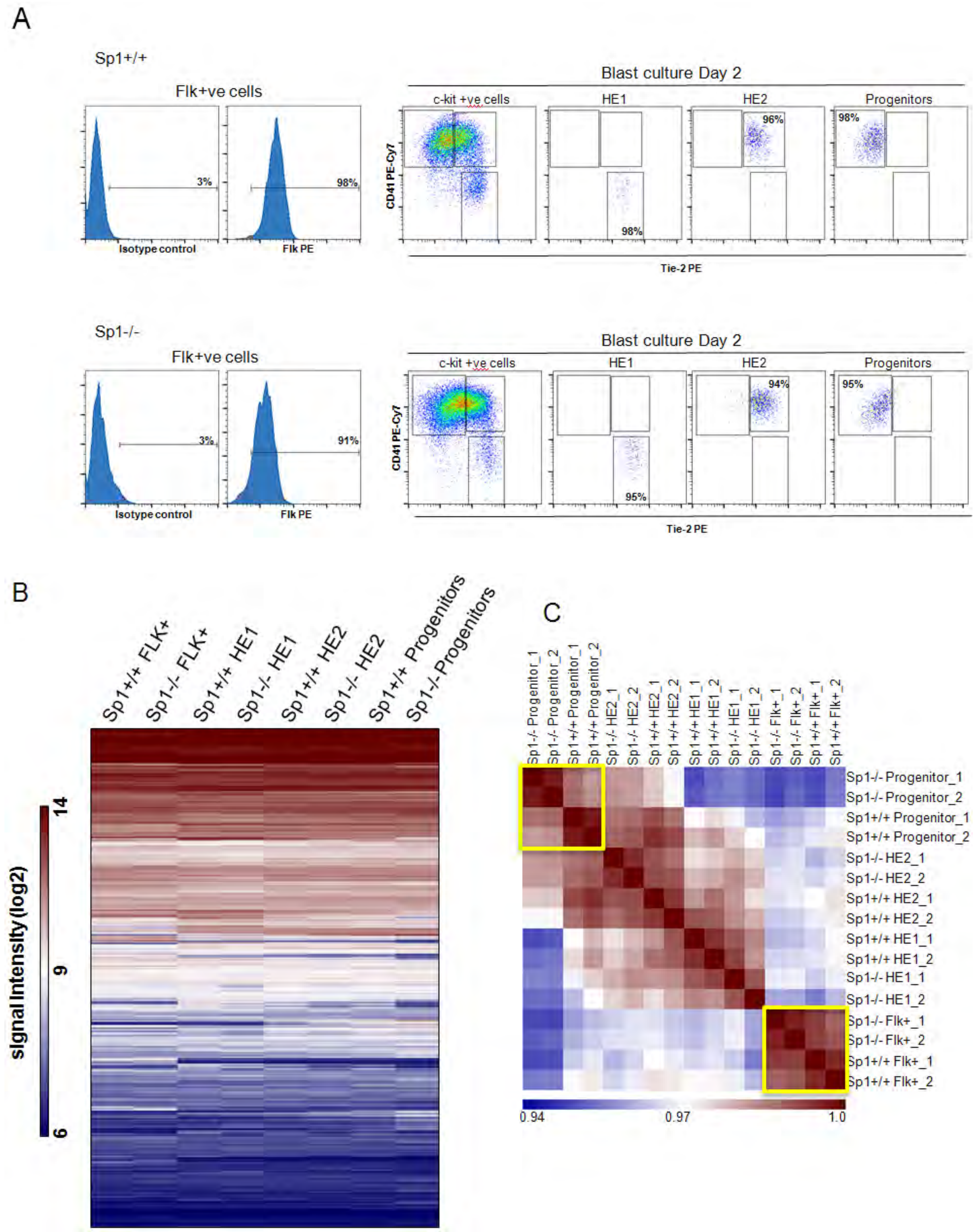


**Supplementary Figure 3.**

**A.** Sorted populations used for expression microarray analysis. Flk1+ve cells were sorted from embryoid bodies cultured in IVD medium for 3.25-3.75 days using MACS columns. FACS histograms show Flk1 staining in Flk1+ve and –ve cell populations. More than 90% of the +ve population stained positive for Flk1. Cells were harvested at Day 2 of blast culture

differentiation, stained with anti KIT, Tie2 and CD41 antibodies and sorted into HE1 (KIT+ve, Tie2+ve, CD41-ve), HE2 (KIT+ve, Tie2+ve, CD41+ve) and progenitor (KIT+ve, Tie2-ve, CD41+ve) cell populations on a Moflow FACS sorter. The sorted populations were more than 95% pure. **B**. Microarray expression analysis of RNA prepared from purified Flk1+ cells, the first and second stage of the hemogenic endothelium and from progenitors (for a detailed description see Figure 2A). Hierarchical clustering of gene expression signals for all genes that are expressed in Sp1+/+ and Sp1-/- cells in the four cell populations. Technical replicates were averaged. **C.** Pearson correlation analysis of the microarray signals obtained with the sorted populations. Duplicates have a strong similarity and cell populations cluster together but show less similarity as differentiation progresses.

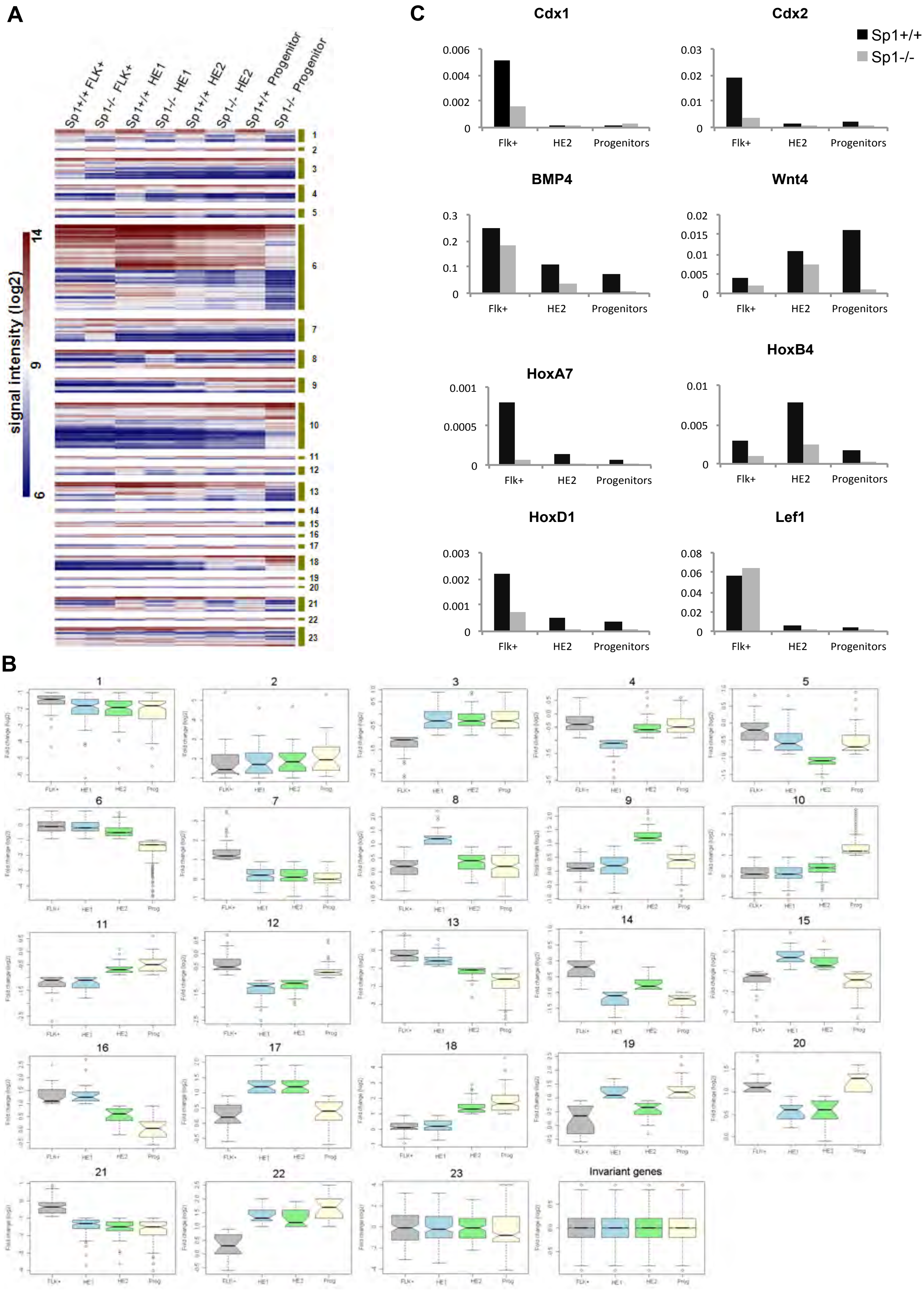
A
Sp1+/+ FLK+
Sp1-/- FLK+
Sp1+/+ HE1
Sp1-/- HE1
Sp1+/+ HE2
Sp1-/- HE2
Sp1+/+ Progenitor
Sp1-/- Progenitor
signal intensity (log2)
14
9
6
1 2 3 4 5 6 7 8 9 10 11 12 13 14 15 16 17 18 19 20 21 22 23
C
Cdx1
Cdx2
Sp1+/+
Sp1-/-
BMP4
Wnt4
HoxA7
HoxB4
HoxD1
Lef1
Flk+
HE2
Progenitors
B
Invariant genes
FLK+
HE1
HE2
Prog

**Supplementary Figure 4.**

**A.** Hierarchical clustering of gene expression signal intensity defining the 23 clusters for each of the populations as depicted in the heat-map. **B**. Box-plots depicting gene expression fold-change of genes within the 23 clusters demonstrating that clustering is valid. **C.** Validation of microarray gene expression was performed for selected genes using RT-PCR.

**Supplementary Figure 5**

A

| | Peaks | All Genes | Expressed Genes |
|---|---|---|---|
| Flk1+ve | 16421 | 11961 | 10165 |
| Progenitors | 8945 | 7611 | 6592 |

B

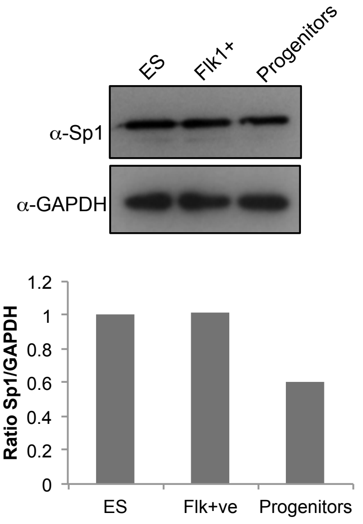


C

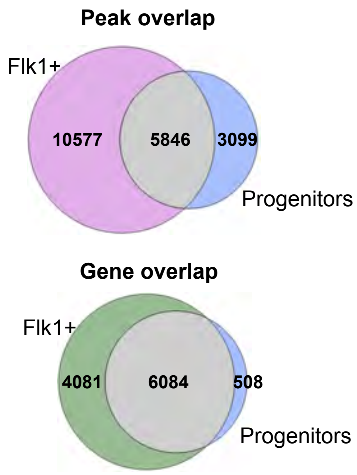


D

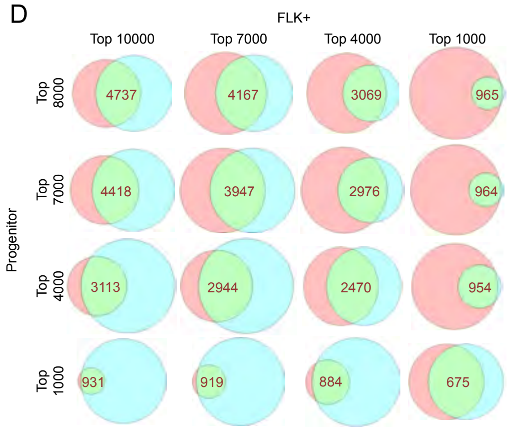


E

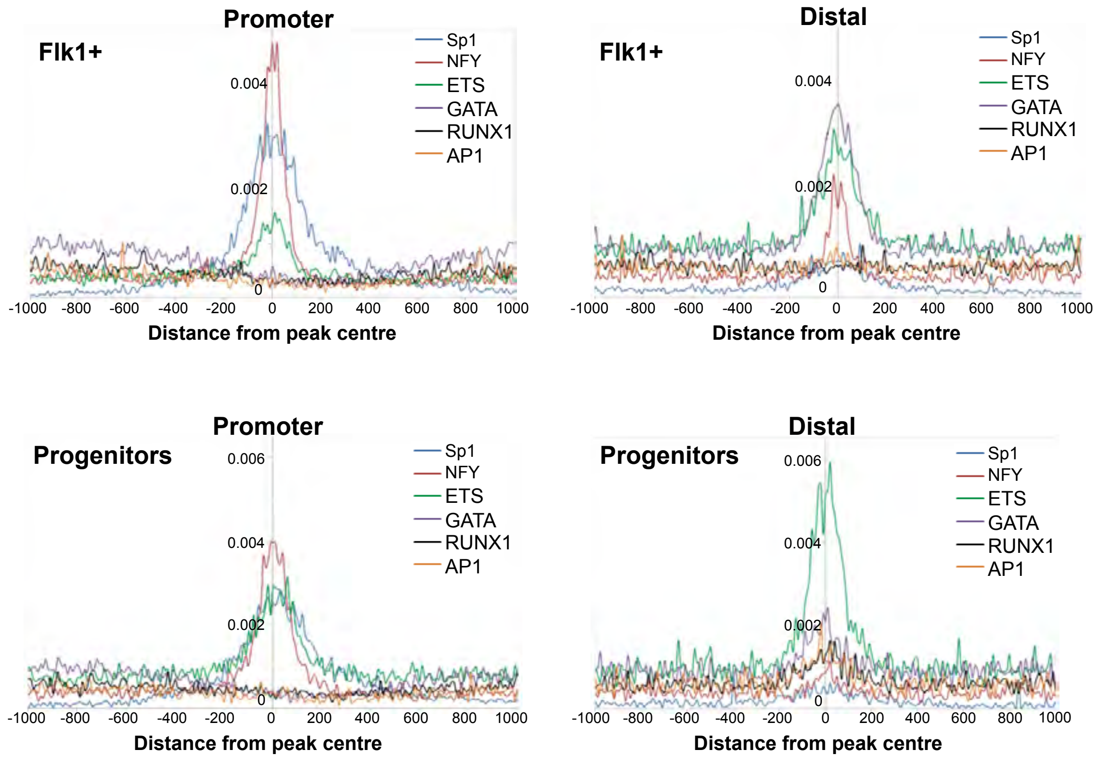

**Supplementary Figure 5 ct'd**

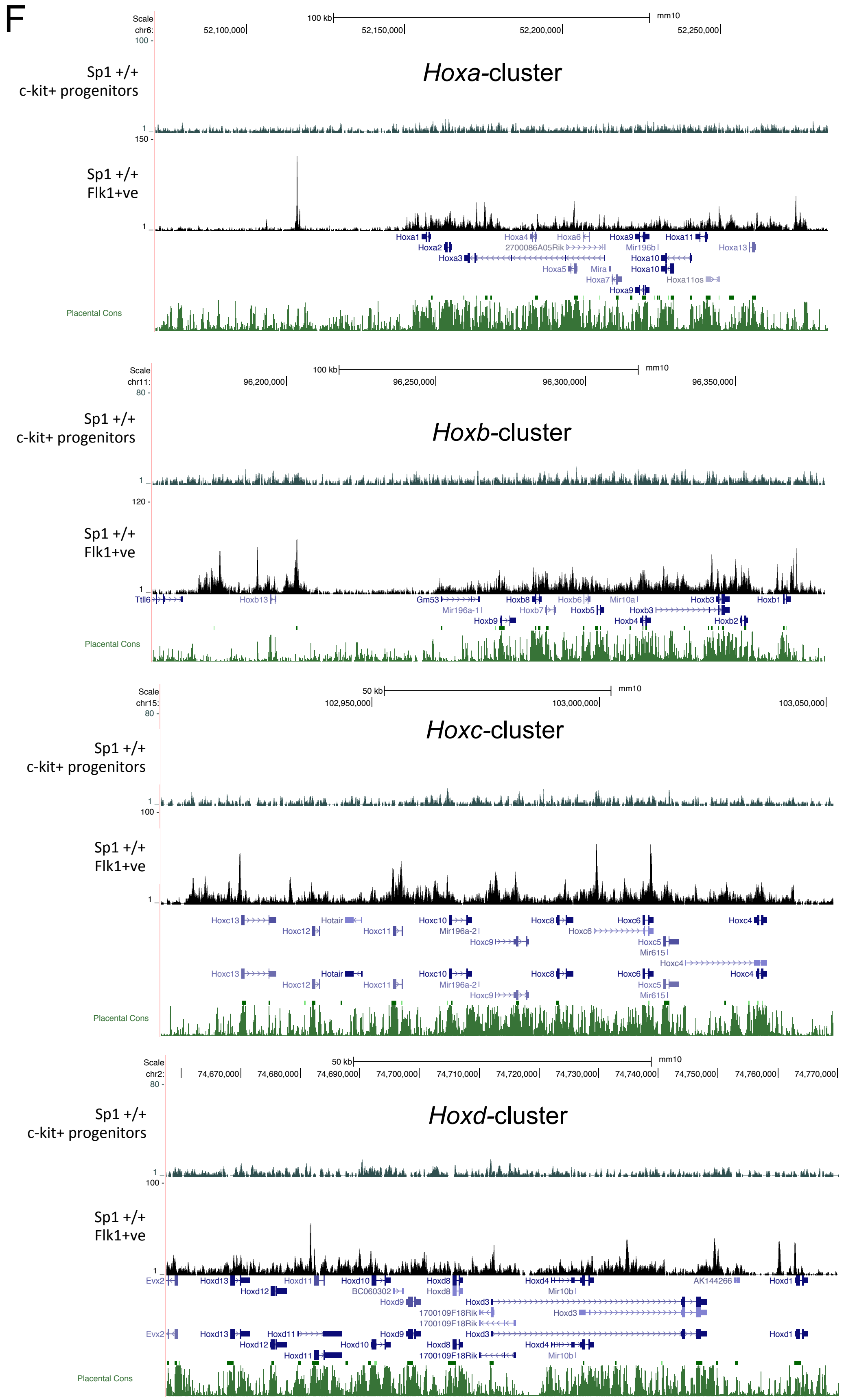

**Supplementary Figure 5.**

**A**. Table showing the numbers of peaks and genes for each cell population. **B**. Western blot showing Sp1 expression in ES, Flk1+ and Progenitor cells. Levels of Sp1 were quantified using Bio-Rad Quantity One Software and normalised to GAPDH. Graph shows an average of two experiments. **C**. Venn diagram showing the overlap in Sp1 peaks (left panel) and genes (right panel) between the Flk1+ve and progenitor populations. **D**. Sp1 binds to an overlapping, but not identical set of targets in Flk1+ve and progenitor cells; Venn diagrams showing the overlap in the binding sites for the top hits in Flk1+ve and progenitors ranked in order of increasing tag-counts. **E**. Motif distribution around the Sp1 binding sites show the positions of the other TF binding motifs that co-localise with Sp1, indicating that most motifs are located within 400 base-pairs of the Sp1 binding site. **F**. Screenshots showing Sp1 binding at the *HoxA, B, C, D* gene clusters.

A

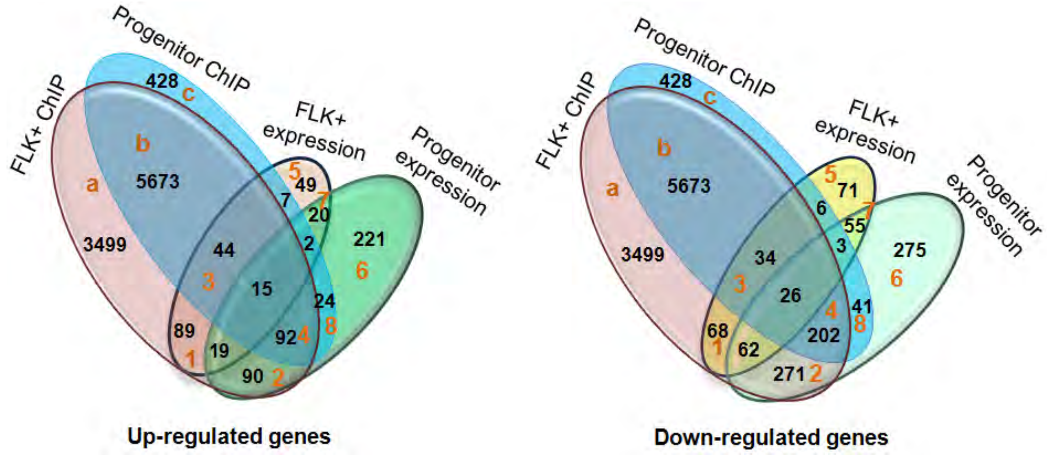
FLK+ ChIP
Progenitor ChIP
FLK+ expression
Progenitor expression
a
b
c
428
5673
3499
49
7
20
2
221
44
15
24
89
19
92
90
1
2
3
4
5
6
7
8
Up-regulated genes
FLK+ ChIP
Progenitor ChIP
FLK+ expression
Progenitor expression
428
5673
3499
71
6
55
3
275
34
26
41
68
62
202
271
Down-regulated genes

B

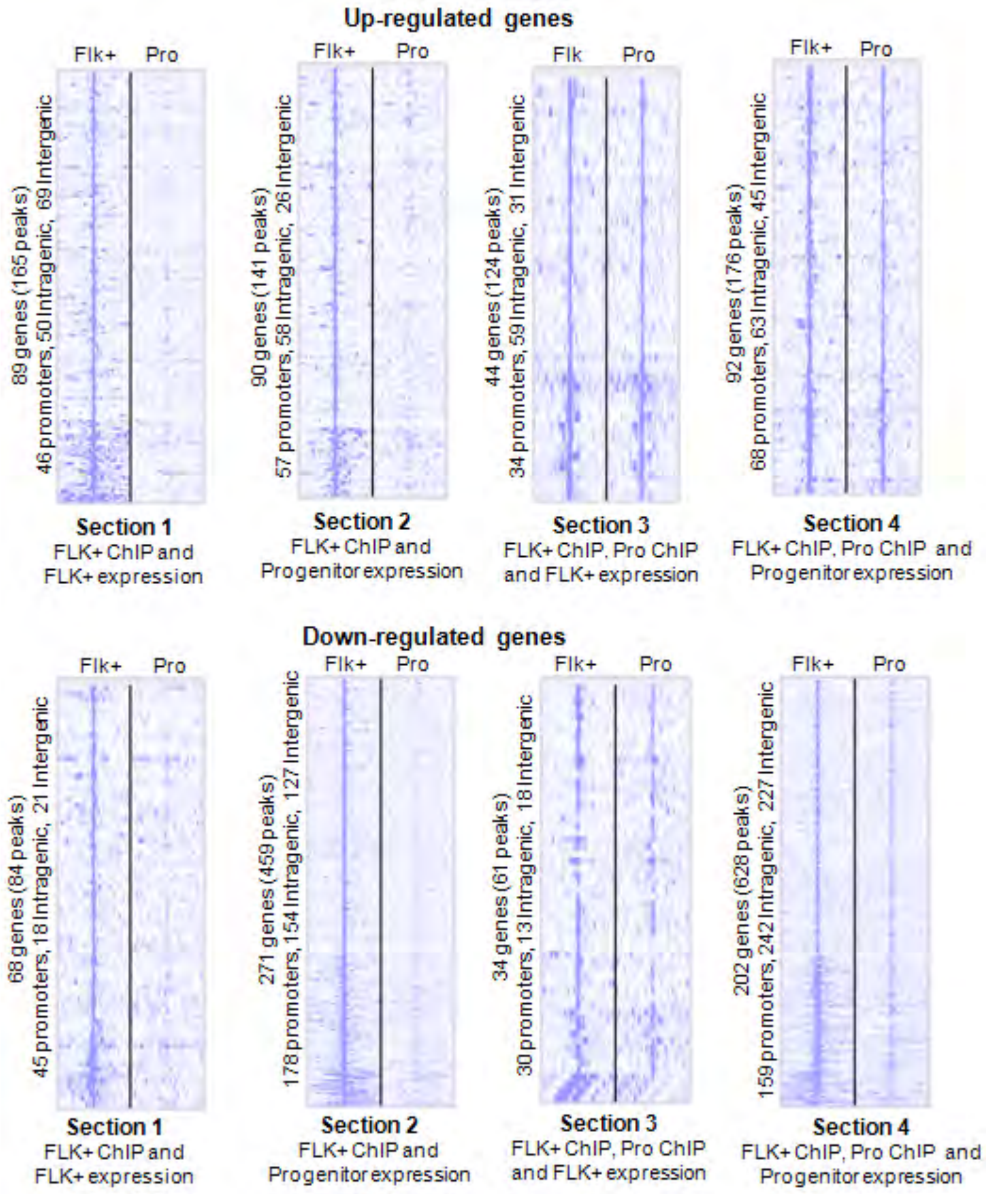
Up-regulated genes
Flk+
Pro
89 genes (165 peaks)
46 promoters, 50 Intragenic, 69 Intergenic
Section 1
FLK+ ChIP and FLK+ expression
90 genes (141 peaks)
57 promoters, 58 Intragenic, 26 Intergenic
Section 2
FLK+ ChIP and Progenitor expression
Flk
44 genes (124 peaks)
34 promoters, 59 Intragenic, 31 Intergenic
Section 3
FLK+ ChIP, Pro ChIP and FLK+ expression
92 genes (176 peaks)
68 promoters, 63 Intragenic, 45 Intergenic
Section 4
FLK+ ChIP, Pro ChIP and Progenitor expression
Down-regulated genes
68 genes (84 peaks)
45 promoters, 18 Intragenic, 21 Intergenic
Section 1
FLK+ ChIP and FLK+ expression
271 genes (459 peaks)
178 promoters, 154 Intragenic, 127 Intergenic
Section 2
FLK+ ChIP and Progenitor expression
34 genes (61 peaks)
30 promoters, 13 Intragenic, 18 Intergenic
Section 3
FLK+ ChIP, Pro ChIP and FLK+ expression
202 genes (628 peaks)
159 promoters, 242 Intragenic, 227 Intergenic
Section 4
FLK+ ChIP, Pro ChIP and Progenitor expression

**Supplementary Figure 6.**

**A**. Venn diagram showing the integration of ChIP-seq and gene expression data in Flk1+ and progenitor cells. The number of genes in sections a, b and c shows the number of genes that are neither up nor down regulated which are the same numbers in the right and left panel. **B**. Heatmaps depicting the distribution of ChIP-seq signals found in genes responsive to Sp1 knock-out around the Sp1 binding sites in Flk1+ cells and progenitors. The labelling on the left indicates the number of genes and the position of the peaks (promoter, intragenic, intergenic)

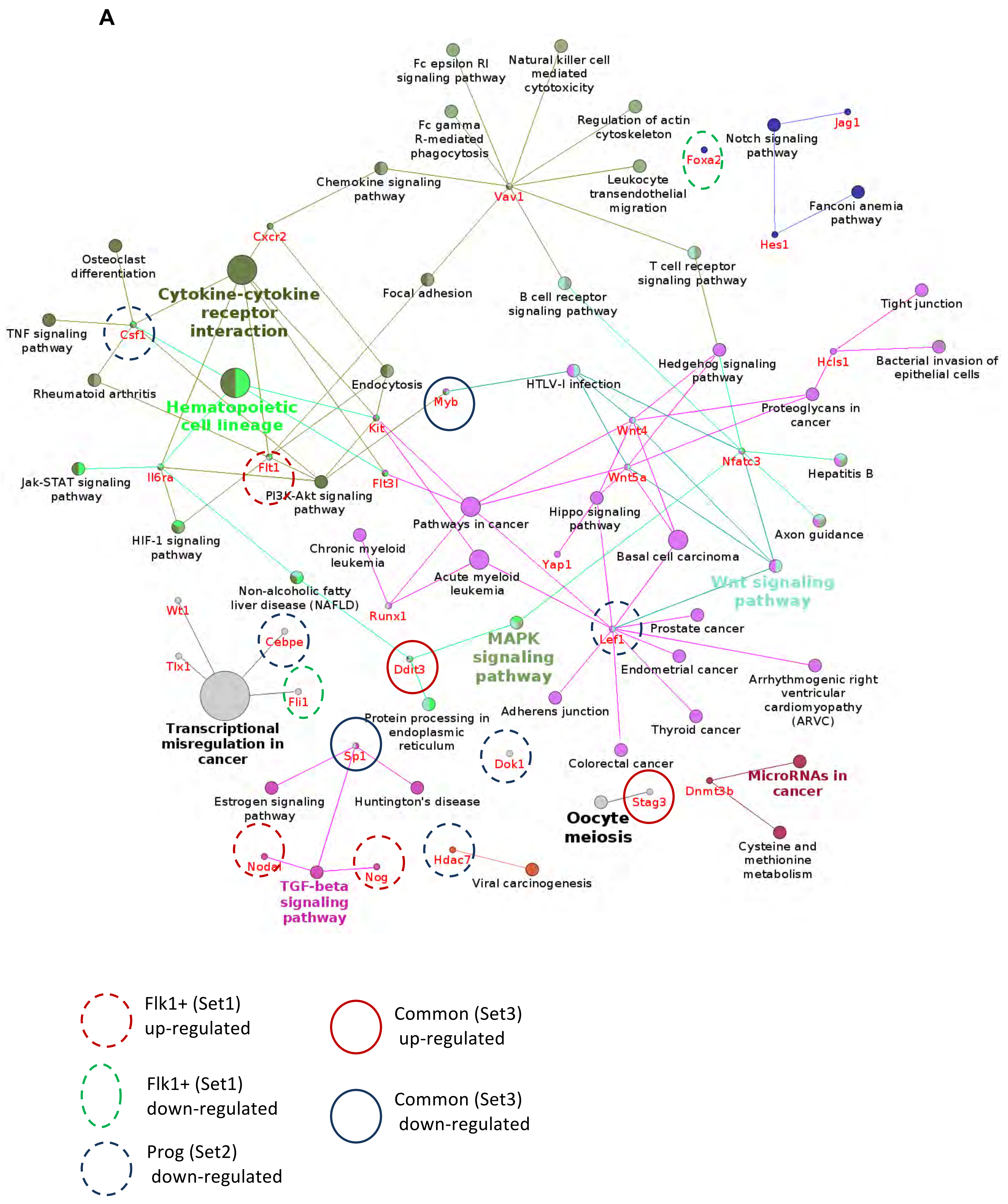


**KEGG pathway analysis of deregulated genes in Sp1-/- Flk1+ cells and progenitors**

**B**

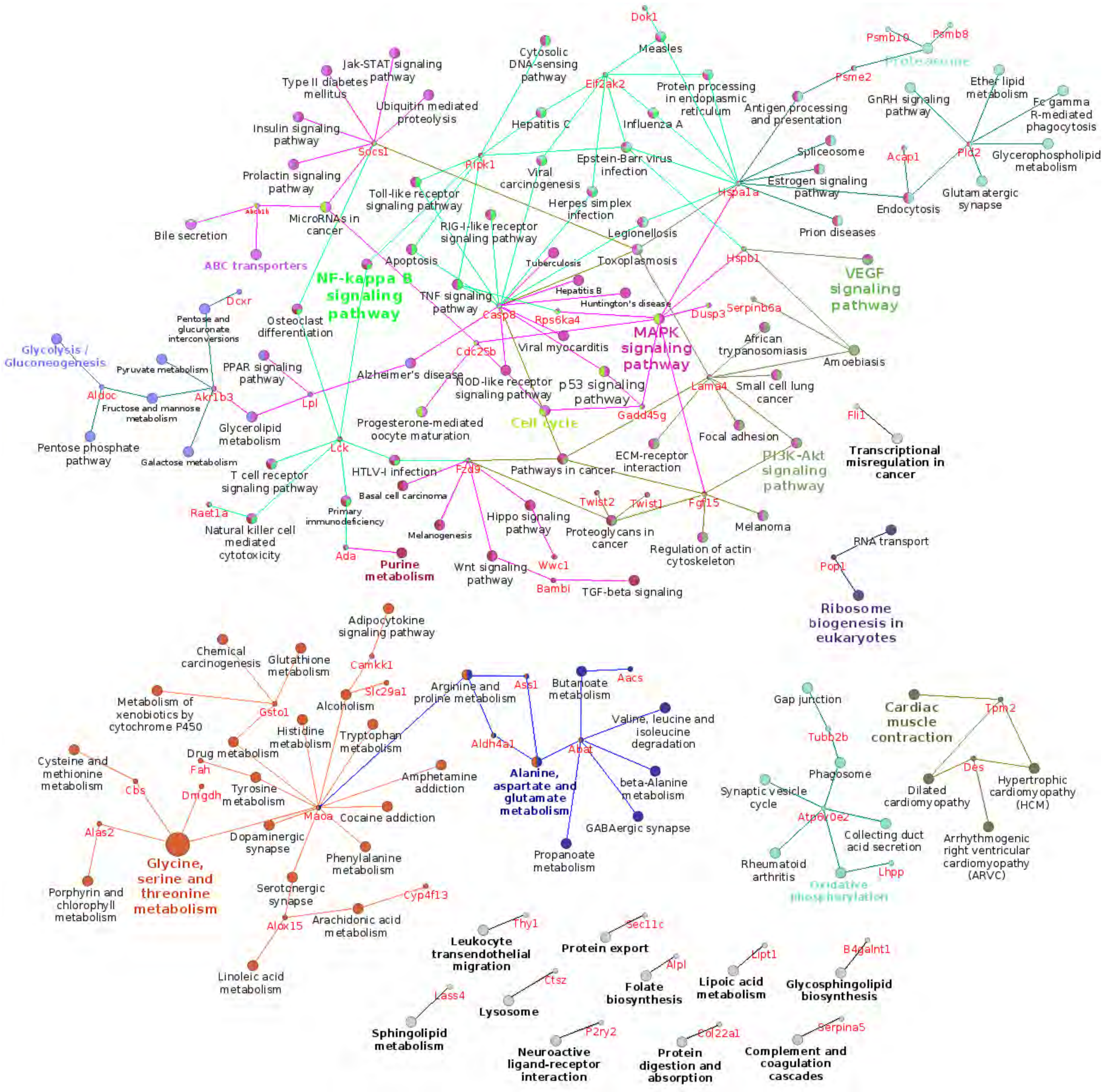


**KEGG pathway analysis of all Sp1 target genes down-regulated in Flk1+ cells**

C

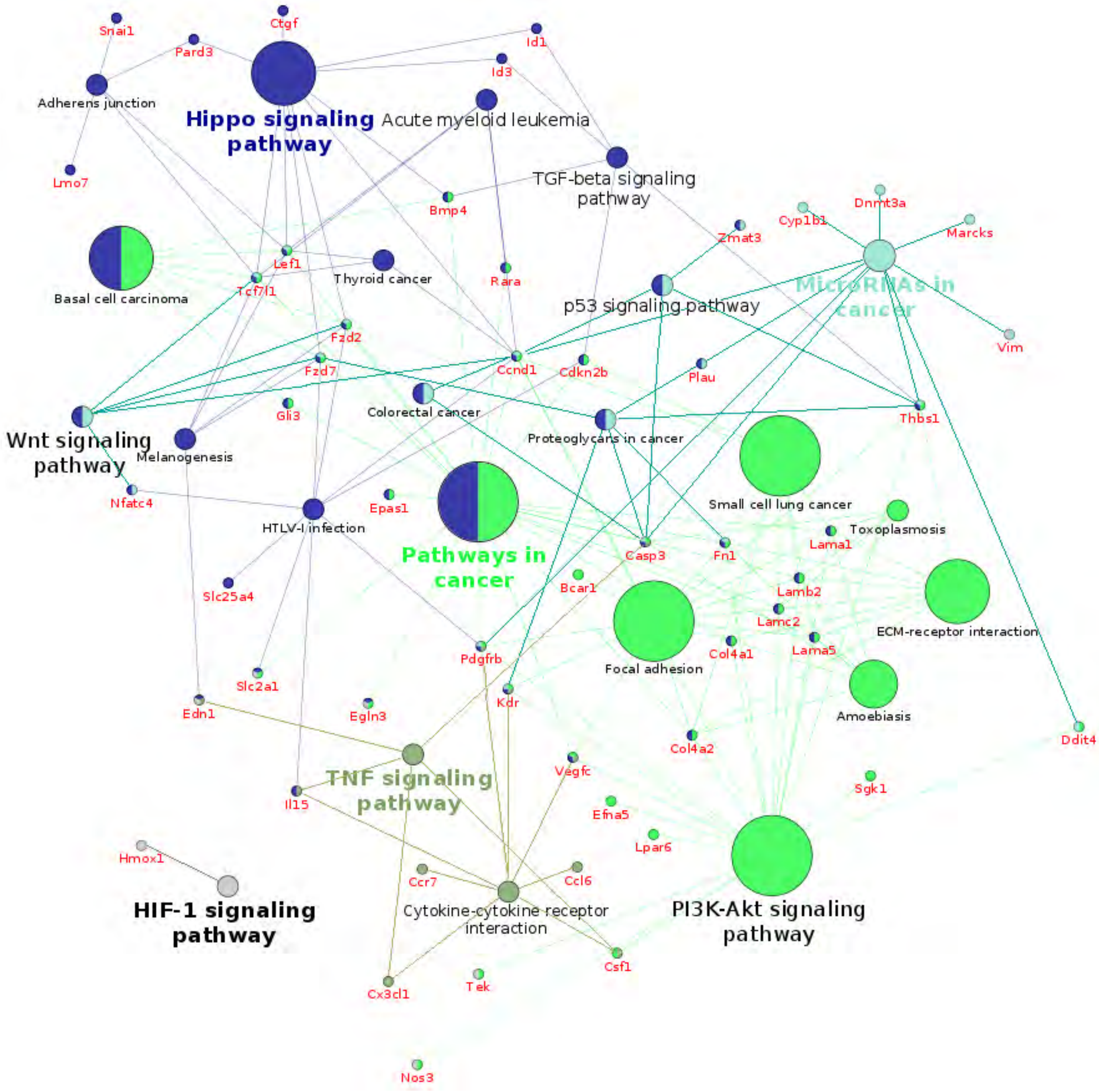


**KEGG pathway analysis of all Sp1 target genes down-regulated in progenitor cells**

D

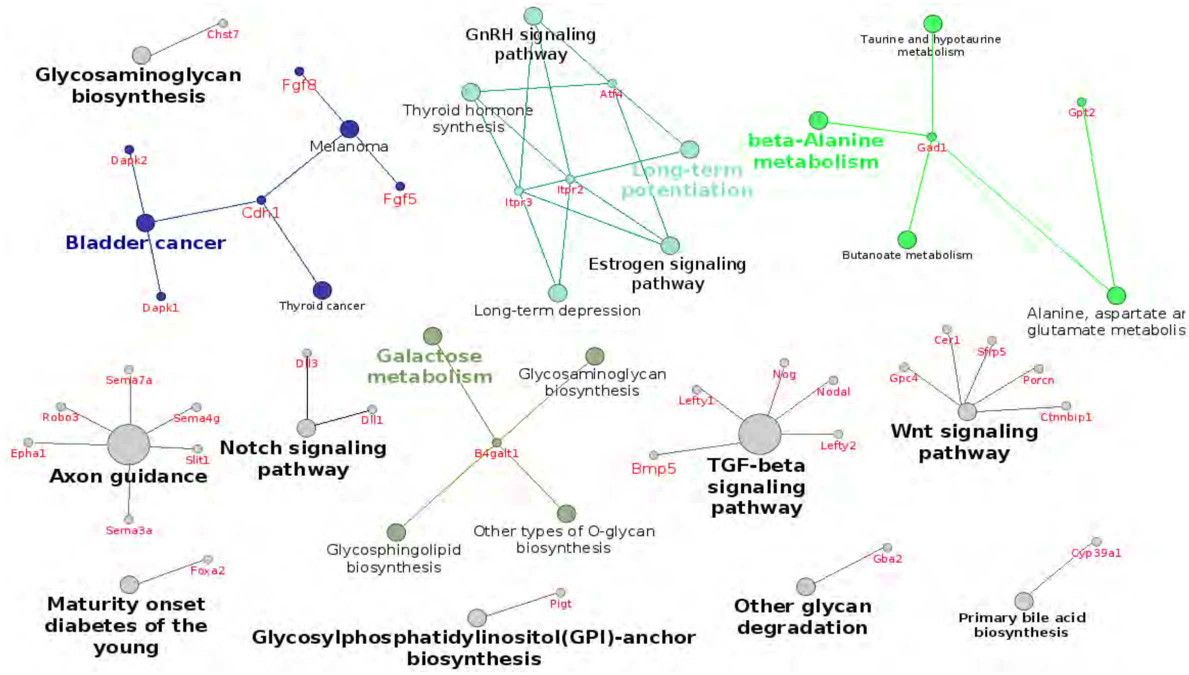


KEGG pathway analysis of all target genes up-regulated in Flk1+ cells

E

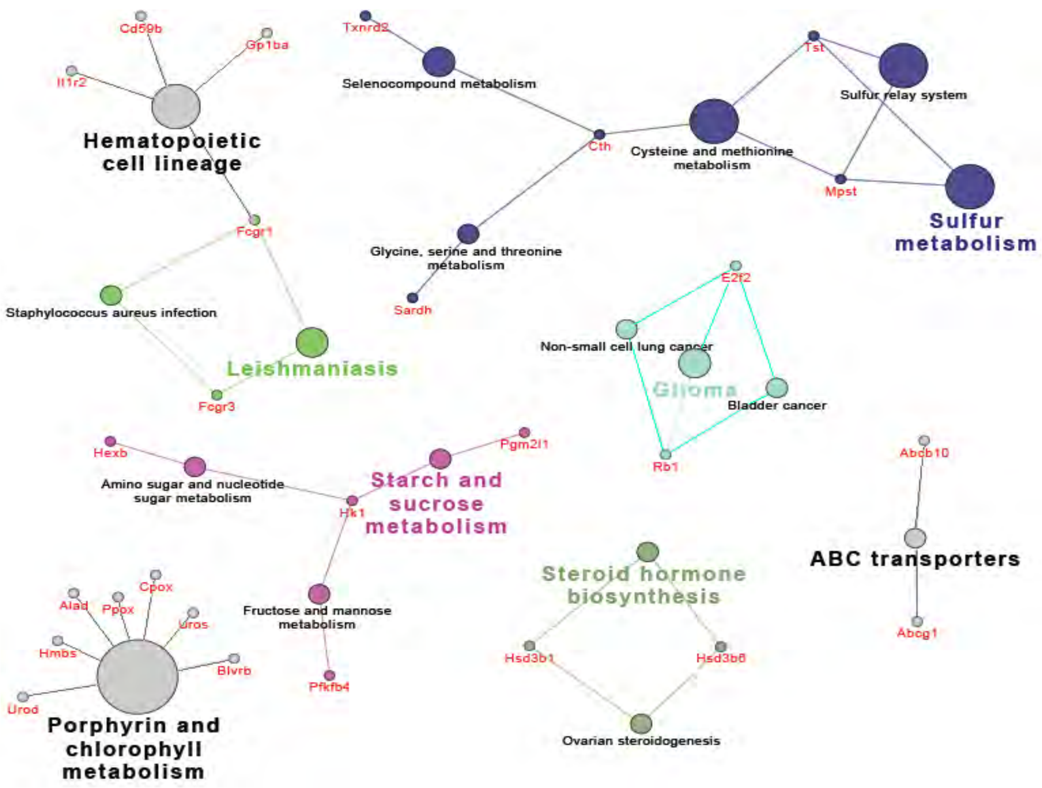


KEGG pathway analysis of all target genes up-regulated in progenitor cells

**Supplementary Figure 7.** KEGG pathway analysis: functionally grouped KEGG pathway term networks using kappa statistics implemented by ClueGO to link the terms in the network. The right-sided enrichment (depletion) test based on the hyper-geometric distribution is used for terms and groups. The groups are created by iterative merging of initially defined groups based on the kappa score threshold. The relationship between the selected terms is defined based on their shared genes and the final groups are randomly coloured where functional groups represented by their most significant term. One, two or more colours represents that a gene/term is a member of one, two or more groups respectively. The size of the nodes reflects the enrichment significance of the terms. The network is automatically laid out using the layout algorithm supported by Cytoscape. **A**: KEGG pathway analysis of selected deregulated genes in Sp1-/- Flk1+ cells and progenitors that are shown in Figure 4C. **B**: KEGG pathway analysis of all Sp1 target genes down-regulated in Flk1+ cells. **C**: KEGG pathway analysis of all Sp1 target genes down-regulated in progenitor cells. **D**: KEGG pathway analysis of all target genes up-regulated in Flk1+ cells. **E**: KEGG pathway analysis of all target genes up-regulated in progenitor cells.

**Download Table S1**

**Download Table S2**

**Download Table S3**

**Download Table S4**

**Download Table S5**
